# Chain entanglement-stabilized injectable zwitterionic hydrogel for vitreous replacement and retinal tamponade

**DOI:** 10.1093/nsr/nwag413

**Published:** 2026-07-06

**Authors:** Haolun Wang, Hongying Wang, Liping Lang, Huijie Hao, Yuqin Cheng, Danyang Chen, Xu Tian, Boshi Liu, Xiaoli Xing, Xiaorong Li, Wenguang Liu, Jianhai Yang

**Affiliations:** School of Materials Science and Engineering, State Key Laboratory of Precious Metal Functional Materials, Tianjin University, Tianjin 300350, China; School of Materials Science and Engineering, State Key Laboratory of Precious Metal Functional Materials, Tianjin University, Tianjin 300350, China; School of Materials Science and Engineering, State Key Laboratory of Precious Metal Functional Materials, Tianjin University, Tianjin 300350, China; School of Materials Science and Engineering, State Key Laboratory of Precious Metal Functional Materials, Tianjin University, Tianjin 300350, China; Tianjin Key Laboratory of Retinal Functions and Diseases, Tianjin Branch of National Clinical Research Center for Ocular Disease, Eye Institute and School of Optometry, Tianjin Medical University Eye Hospital, Tianjin 300384, China; School of Materials Science and Engineering, State Key Laboratory of Precious Metal Functional Materials, Tianjin University, Tianjin 300350, China; School of Materials Science and Engineering, State Key Laboratory of Precious Metal Functional Materials, Tianjin University, Tianjin 300350, China; School of Materials Science and Engineering, State Key Laboratory of Precious Metal Functional Materials, Tianjin University, Tianjin 300350, China; Tianjin Key Laboratory of Retinal Functions and Diseases, Tianjin Branch of National Clinical Research Center for Ocular Disease, Eye Institute and School of Optometry, Tianjin Medical University Eye Hospital, Tianjin 300384, China; Tianjin Key Laboratory of Retinal Functions and Diseases, Tianjin Branch of National Clinical Research Center for Ocular Disease, Eye Institute and School of Optometry, Tianjin Medical University Eye Hospital, Tianjin 300384, China; Tianjin Key Laboratory of Retinal Functions and Diseases, Tianjin Branch of National Clinical Research Center for Ocular Disease, Eye Institute and School of Optometry, Tianjin Medical University Eye Hospital, Tianjin 300384, China; School of Materials Science and Engineering, State Key Laboratory of Precious Metal Functional Materials, Tianjin University, Tianjin 300350, China; School of Materials Science and Engineering, State Key Laboratory of Precious Metal Functional Materials, Tianjin University, Tianjin 300350, China

**Keywords:** chain entanglement, zwitterionic hydrogel, vitreous substitute, antifouling, injectability

## Abstract

Vitreoretinal diseases often require vitrectomy followed by intraocular tamponade to stabilize the retina. Although commonly used expansile gases and silicone oil (SO) can promote retinal reattachment, long-term vitreous replacement remains limited by postural requirements and postoperative complications, including inflammation and SO emulsification. Here, we report an injectable poly(2-methacryloyloxyethyl phosphorylcholine) (PMPC) zwitterionic hydrogel stabilized by topological chain entanglements as a vitreous substitute with clinical translational potential. The PMPC hydrogel is delivered via minimally invasive shear-thinning injection and demonstrates favorable long-term intraocular tolerability and biocompatibility in a rabbit vitrectomy model. We further assess its tamponade-relevant performance in a rabbit rhegmatogenous retinal detachment model by examining its ability to maintain break coverage and support sustained retinal reattachment. Importantly, this PMPC hydrogel is built from a clinically validated zwitterionic polymer with established industrial supply and regulatory precedent, which may reduce manufacturing and regulatory complexity and facilitate earlier clinical evaluation as a practical alternative to SO.

## Teaser

An injectable, crosslinker-free PMPC zwitterionic hydrogel stabilized by topological entanglements combines optical clarity, antifouling, biocompatibility, retinal break sealing, sustained reattachment and translational potential as an SO alternative.

## INTRODUCTION

Many vitreoretinal disorders, such as retinal detachment (RD, including rhegmatogenous, exudative and tractional forms), macular hole, proliferative vitreoretinopathy (PVR), diabetic retinopathy and vitreous hemorrhage, often require pars plana vitrectomy, followed by intraocular tamponade to stabilize the retina and maintain ocular integrity [1–4]. Currently used tamponade agents, including expanding gases and silicone oil (SO), can effectively promote postoperative retinal reattachment, yet they still fall short of the requirements for long-term stability and biosafety. Postoperative complications, such as cataract formation, elevated intraocular pressure (IOP), intraocular inflammation and SO emulsification, remain relatively common [5–7].

Compared with conventional tamponade materials, injectable hydrogels have attracted increasing interest as vitreous substitutes because of their high water content, tunable viscoelasticity and optical and physicochemical properties that more closely resemble those of the natural vitreous [8–11]. Moreover, injectable hydrogels can be delivered through small-gauge needles in a minimally invasive manner, and their shear-thinning rheology enables facile injection while allowing the material to recover a gel-like state after extrusion within the vitreous cavity. However, vitreous substitutes are intended for prolonged intraocular residence. Once proteins deposit and cells adhere at the material interface, persistent inflammation and foreign-body responses may ensue, potentially leading to fibrosis, interfacial instability and retina-related complications that compromise surgical outcomes. Accordingly, zwitterionic polymers have been increasingly explored to create highly hydrated interfaces that suppress protein adsorption and cell adhesion, thereby reducing biofouling and immune-related risks associated with long-term intraocular implantation [12–15].

These injectable zwitterionic hydrogel systems, including copolymer-based designs, chemically crosslinked networks and newly developed zwitterionic monomers, have demonstrated the functional benefits as vitreous substitutes [16–20]. Nevertheless, many emerging systems still face practical barriers to clinical translation, such as scalable raw-material supply, batch-to-batch quality control, comprehensive long-term *in vivo* safety validation and a clearly navigable regulatory pathway. In contrast, zwitterionic building blocks with established industrial supply chains and prior regulatory precedents are particularly attractive for developing practical alternatives to clinically used tamponades, such as SO. A representative example is poly(2-methacryloyloxyethyl phosphorylcholine) (PMPC), whose cell-membrane-mimetic phosphorylcholine side chains enable PMPC to form a strongly hydrated interface that suppresses non-specific protein deposition and subsequent cell attachment, while also offering tangible engineering and regulatory credibility [13,21–23]. PMPC surface coatings have been implemented in approved medical devices, exemplified by the coronary stent BiodivYsio AS®, which received US Food and Drug Administration (FDA) approval in 2000. In addition, a phosphorylcholine–butyl methacrylate copolymer has been registered with the FDA under the trade name Lipidure®-CM5206, supporting its use as a device-grade material [21]. Moreover, 2-methacryloyloxyethyl phosphorylcholine (MPC)-containing soft hydrogel contact lenses (e.g., Proclear and Omafilcon A) have been commercialized and used in ophthalmic products worldwide, providing further evidence for the long-term ocular biocompatibility of this material class [24,25]. However, traditional PMPC hydrogels, which rely on copolymerization or chemical crosslinking, often exhibit high brittleness and poor injectability [26–29]. Moreover, after intraocular injection, they typically struggle to rapidly re-establish a hydrogel network capable of supporting the retina, which may lead to insufficient IOP during surgery and hinder accurate assessment of the required injection volume.

**Scheme 1.**
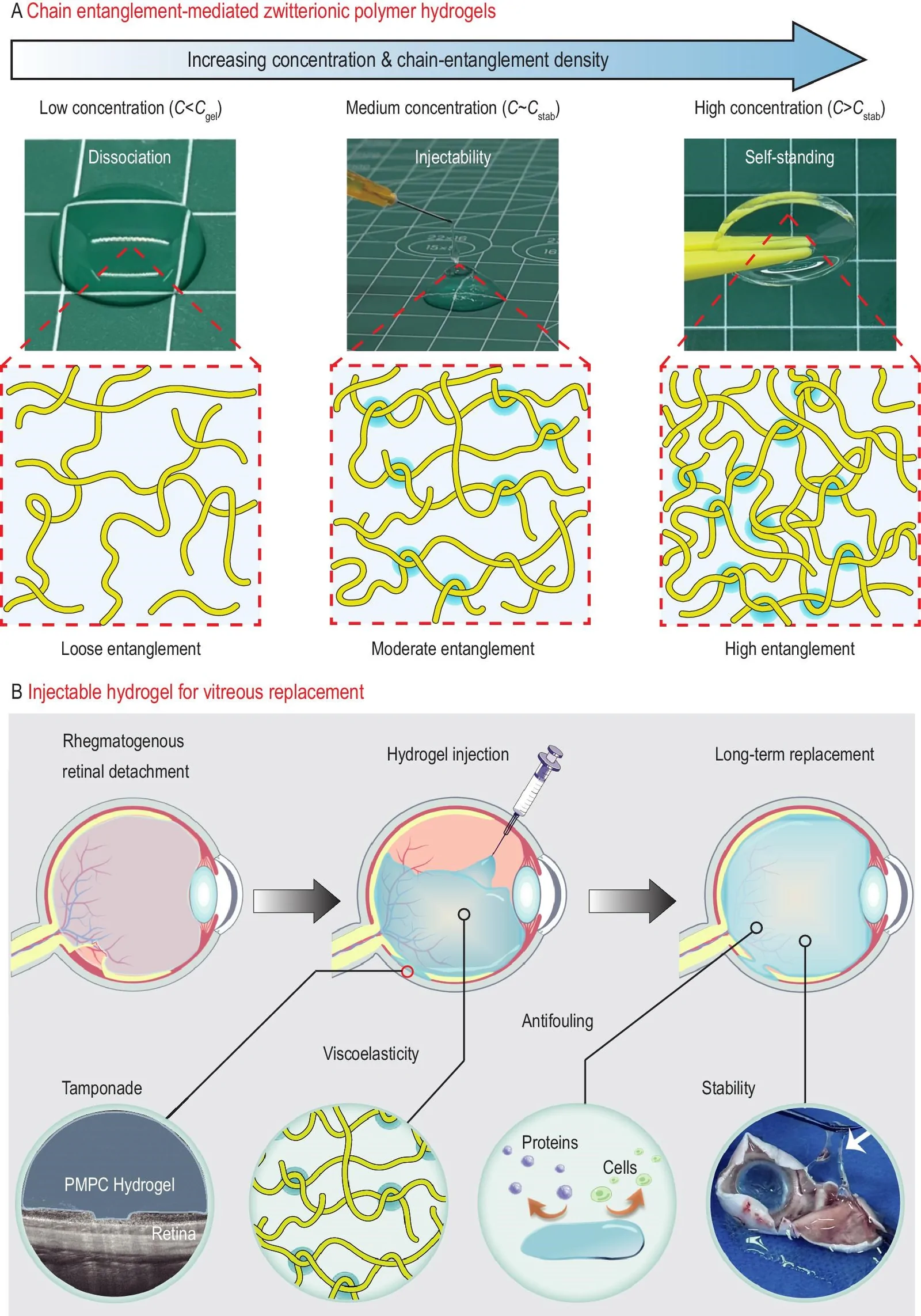
Chain entanglement-stabilized injectable zwitterionic hydrogel for vitreous replacement and retinal tamponade. (A) Schematic illustration of chain entanglement-mediated zwitterionic polymer hydrogels. With increasing monomer concentration, chain entanglement density transitions from loose to high, resulting in enhanced mechanical properties ranging from dissociation to injectability and self-standing integrity. (B) Schematic of injectable zwitterionic polymer hydrogel application for vitreous replacement. Following RD, the shear-thinning hydrogel is injected to provide tamponade, offering vitreous-like viscoelasticity, antifouling properties and long-term intraocular stability.

Herein, we present a minimalist injectable hydrogel design for vitreous substitution, in which a macroscopic PMPC network forms without external crosslinkers or deliberately engineered associative motifs; instead, mechanical integrity is primarily governed by topological chain entanglement (Scheme 1). From a polymer-physics perspective, chain entanglement-driven gelation is promoted by two key factors: sufficiently long polymer chains and sufficiently high polymer/monomer concentration, both of which increase interchain interpenetration and strengthen topological constraints against chain disengagement. Accordingly, we employ an ultralow photoinitiator (0.1 mol% relative to the monomer) loading to favor the growth of long PMPC chains [30–36], and we systematically vary MPC concentration over a wide range to map the critical gelation threshold as well as the concentration window required to maintain swelling-stable integrity under physiological hydration. This enables the hydrogel to undergo physiological swelling without structural disintegration after minimally invasive intraocular injection, thereby preserving its macroscopic integrity and functional performance as a vitreous substitute. With this chain entanglement-governed network formation strategy, we show that the PMPC hydrogel supports long-term vitreous replacement in a standard vitrectomy model with longitudinal follow-up to 6 months, and we further examine its tamponade-relevant performance in a rhegmatogenous RD model by assessing its ability to bridge retinal breaks. Collectively, this crosslinker-free and formulation-simplified approach relies on topological chain entanglement to stabilize the fully hydrated gel network, while employing a clinically validated zwitterionic polymer; together, these features may reduce manufacturing and regulatory complexity and thereby facilitate earlier clinical evaluation as a practical alternative to SO.

## RESULTS AND DISCUSSION

### Topological gelation of PMPC hydrogels

**Figure 1.**
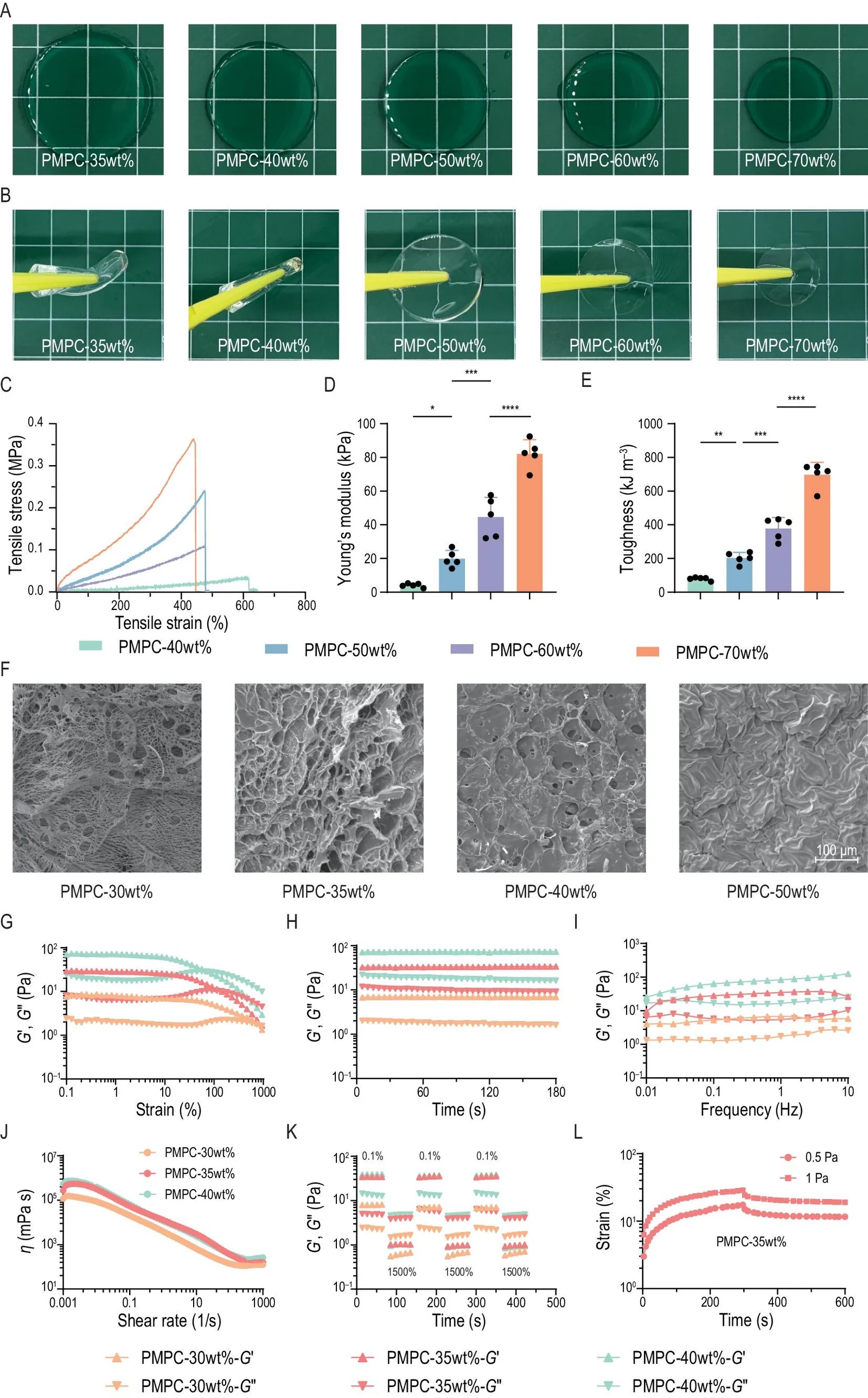
Topological gelation of PMPC hydrogels. (A and B) PMPC hydrogel discs (35–70 wt%) after 7 days of swelling equilibrium in PBS. Photographs showing stable macroscopic integrity in the fully hydrated state (A) and demonstration of hydrogels held with forceps (B). (C–E) Tensile stress–strain curves of as-prepared PMPC hydrogels at monomer concentrations of 40–70 wt% (C), with the corresponding Young’s modulus (D) and toughness (E). *P* values were calculated by one-way analysis of variance (ANOVA) followed by Tukey’s post hoc test. *P < 0.05, **P < 0.01, ***P < 0.001, ****P < 0.0001. (F) SEM images of PMPC hydrogels prepared at 30–50 wt% (scale bar, 100 μm). (G) Strain-sweep analysis of PMPC hydrogels over a strain range of 0.1%–1000%. (H) Time-sweep curves of PMPC hydrogels measured at 1 Hz frequency and 0.1% strain over a duration of 180 s. (I) Frequency sweep curves of PMPC hydrogels in a frequency range from 0.01 to 10 Hz at 0.1% strain. (J) Viscosity profile of PMPC hydrogels across a shear rate ranging from 0.001 to 1000 s^−1^. (K) The variation in *G*′ and *G*″ values of PMPC hydrogels was measured by alternating between small strain (γ = 0.1%) and large strain (γ = 1500%) at a fixed angular frequency (1 Hz). Each strain interval was kept at 100 s. (L) Strain curves of PMPC hydrogels plotted as a function of time in the shear creep test under different applied shear stresses (0.5 and 1.0 Pa). All tests were performed at least three times at 37°C.

We systematically varied the concentration of the MPC monomer from 20 to 70 wt% to identify the minimum gelation concentration (*C*_gel_) and the minimum concentration required for swelling-stable macroscopic integrity (*C*_stab_). As shown in Fig. S1A, the 20 wt% precursor did not form a macroscopic hydrogel after photopolymerization and remained a viscous liquid. At 25 wt%, the sample gelled initially but gradually disintegrated during swelling in phosphate-buffered saline (PBS), indicating that the entanglement density was insufficient to withstand swelling-induced stress and maintain network integrity. In contrast, 30 wt% was the lowest concentration that remained intact after 7 days of swelling, although the resulting hydrogel was highly compliant and not self-supporting during handling (Fig. S1B). With further increases in solid content, the entanglement network strengthened markedly: hydrogels prepared at 50–70 wt% readily formed self-standing structures in the fully hydrated state (Fig. 1A and Fig. S1B). Correspondingly, the equilibrium swelling ratio decreased monotonically with increasing monomer concentration (Fig. 1B and Fig. S2), consistent with a progressively densified entanglement network that more effectively suppresses swelling-driven chain disengagement. It is worth noting that the gel size changed very little between day 1 and day 3 (Fig. S3), indicating that swelling approached a near-plateau within 24 h under these conditions.

The concentration-dependent reinforcement of the entanglement network is also evident in the mechanical response. As shown in Fig. 1C–E, tensile testing revealed a pronounced increase in both stiffness and energy dissipation with increasing MPC concentration: from 40 to 70 wt%, the Young’s modulus of hydrogels increased from 4.1160 to 82.1636 kPa, while the toughness rose from 79.7439 to 697.4823 kJ/m^3^. This trend is consistent with a higher polymer volume fraction and entanglement density at higher concentration, where entanglements act as physical crosslinks to facilitate load transfer and enhance energy dissipation, resulting in concurrent increases in modulus and toughness. Tensile measurements require specimens that remain intact during gripping and stretching; PMPC-35wt% hydrogel samples were too compliant to yield reproducible tensile data, whereas PMPC-40wt% hydrogel samples, although still relatively weak, could be tested to obtain quantitative mechanical metrics. Consistent with these macroscopic trends, SEM images reveal a progressively more compact microstructure as the concentration increases from 30 to 50 wt% (Fig. 1F), supporting strengthened effective entanglement constraints at higher concentrations.

For vitreous substitution, viscoelasticity and injectability are key design targets: the material should provide sufficient elastic support to help stabilize intraocular tissues while retaining a viscous component to dissipate transient mechanical perturbations. Considering that PMPC-50wt% hydrogel and above are mechanically robust yet may be overly stiff for vitreous replacement, we therefore focused rheological characterization on the 30–40 wt% window to guide selection of a formulation that balances swelling-stable integrity, vitreous-comparable viscoelasticity and injectability. Strain-sweep measurements show that PMPC-30wt%, PMPC-35wt% and PMPC-40wt% exhibit solid-like behavior at small deformations, with the storage modulus (*G*′) exceeding the loss modulus (*G*″) within the low-strain regime (0.1%–100%), indicating the presence of a stable three-dimensional network (Fig. 1G). Upon exceeding a critical strain, *G*′ decreases sharply while *G*″ increases, consistent with strain-induced network disruption. Accordingly, a strain amplitude of 1% within the linear viscoelastic region was used for subsequent dynamic tests. Differences in modulus among the three formulations reflect the concentration-dependent strengthening of entanglement constraints. Time-sweep experiments further show that both *G*′ and *G*″ remain stable over the measurement duration (180 s), indicating a steady viscoelastic response under quiescent conditions (Fig. 1H). As shown in Fig. 1I, there is a modest increase in both moduli over 0.1–10 Hz, while *G*′ consistently dominates *G*″ across the tested range, supporting a predominantly elastic viscoelastic character over relevant timescales. Such frequency-dependent behavior suggests that hydrogels can provide mechanical damping and support under dynamic intraocular loading. As an injectable vitreous substitute, the hydrogel should flow readily under high shear during intraocular injection while remaining sufficiently viscous at rest to maintain its position and provide mechanical support in the vitreous cavity. As shown in Fig. 1J, the apparent viscosity decreases markedly with increasing shear rate, demonstrating a typical shear-thinning behavior. This response is advantageous because it reduces flow resistance at high shear (e.g., extrusion through a 25-G needle), while preserving higher viscosity at low shear to promote post-injection shape/positional stability in the vitreous cavity. Mechanical recoverability after large deformation was evaluated by step-strain cycling at 1 Hz, alternating between low strain (γ = 0.1%) and high strain (γ = 1500%). As depicted in Fig. 1K, at γ = 0.1%, *G*′ remained consistently higher than G″, and both moduli were stable over time, indicating a predominantly solid-like response of the entanglement-supported network. When the strain was increased to γ = 1500%, *G*′ dropped below *G*″, reflecting shear-induced disruption of the network under large deformation. Importantly, upon returning to the low-strain condition, the moduli recovered rapidly and reproducibly over multiple cycles, consistent with a reversible physical network that supports injectability and post-deformation recovery. To further probe viscoelastic behavior relevant to vitreous substitution, we performed creep-recovery measurements. Under an applied stress, the hydrogel exhibited time-dependent deformation, and upon load removal, it showed an immediate elastic recoil followed by gradual recovery with a small residual strain (Fig. 1L). These results confirm the viscoelastic nature of the PMPC hydrogel. Its response lies between that of an ideal elastic solid and a purely viscous liquid, which is desirable for maintaining globe geometry under sustained loads and for absorbing transient mechanical impacts. Collectively, the data from strain sweeps, frequency sweeps, shear-thinning and self-healing measurements demonstrate that the PMPC hydrogel possesses viscoelastic properties closely matching those of the natural vitreous.

### Physical properties of PMPC hydrogels

PMPC hydrogels were designed to approximate key physicochemical properties of the native vitreous. We therefore systematically quantified density, refractive index, surface tension, optical transmittance and molecular transport, in parallel with SO, a clinically used vitreous substitute, for direct comparison. As shown in Fig. S4A, the densities of PMPC-30wt%, PMPC-35wt% and PMPC-40wt% were 1.007, 1.011 and 1.015 g/cm^3^, respectively, all higher than that of SO (0.970 g/cm^3^). A density modestly above that of SO is advantageous for intraocular use because it favors gravitational settling in the vitreous cavity rather than buoyant floating after injection. This property may help alleviate the need for strict postoperative posturing in certain scenarios, although clinical positioning ultimately depends on the location of retinal breaks and tamponade conditions. The refractive indices of PMPC-30wt%, PMPC-35wt% and PMPC-40wt% were 1.3375, 1.3372 and 1.3384, respectively, which are close to the reported range for the native vitreous (1.3345–1.3348) (Fig. S4B and Table S1). In contrast, SO exhibits a substantially higher refractive index (1.4040). Such refractive mismatch can induce refractive shifts and optical aberrations, potentially compromising visual quality. By better matching the native vitreous, PMPC hydrogels may help reduce optical discontinuities along the ocular light path. Beyond optical matching, interfacial mechanics are also critical, particularly for rhegmatogenous RD, where effective tamponade requires sustained coverage of retinal breaks to limit fluid ingress and support reattachment. The surface tension values of PMPC-30wt%, PMPC-35wt% and PMPC-40wt% were 20.99, 39.65 and 40.81 mN/m, respectively, compared with 20.64 mN/m for SO under identical measurement conditions (Fig. S4C). Notably, PMPC-35wt% and PMPC-40wt% exhibited markedly higher surface tension than SO, indicating a greater propensity to maintain a continuous interface after injection. In line with this interfacial profile, PMPC hydrogels provided stable coverage of retinal breaks and supported retinal reattachment in our rhegmatogenous model without apparent migration. Collectively, the combination of near-vitreous refractive index and favorable interfacial properties supports the suitability of PMPC hydrogels as vitreous substitutes for break-associated pathology.

Optical performance is a fundamental requirement for vitreous substitutes. PMPC hydrogels were highly transparent, maintaining high optical transmittance across the visible range (400–800 nm). A slight decrease in transmittance with increasing solid content was observed, which is consistent with a progressively densified entanglement-supported network that increases light scattering to a limited extent (Fig. S4D). To better reflect the clinical injection process, we further measured optical transmittance before and after extrusion through a 25-G needle (Fig. S4E), confirming that the injection procedure does not compromise transparency. Beyond optical clarity, an ideal vitreous substitute should permit diffusion and exchange of small molecules, as the native vitreous supports the transport of nutrients, oxygen and metabolic waste products, which are essential for retinal homeostasis. We, therefore, evaluated molecular transport in PMPC hydrogels using sodium fluorescein (molecular weight 365 Da) as a tracer. Sodium fluorescein rapidly diffused into the hydrogel and permeated the matrix within 1 h. We further monitored tracer efflux to assess exchange capability, and fluorescein exited the hydrogel within ∼6 h (Fig. S4F). In contrast, SO exhibited markedly reduced permeability under the same conditions (Fig. S5). Together, these results indicate that PMPC hydrogels combine high optical transparency with efficient small-molecule transport, both of which are desirable for vitreous replacement.

### Antifouling performance of PMPC hydrogels

**Figure 2.**
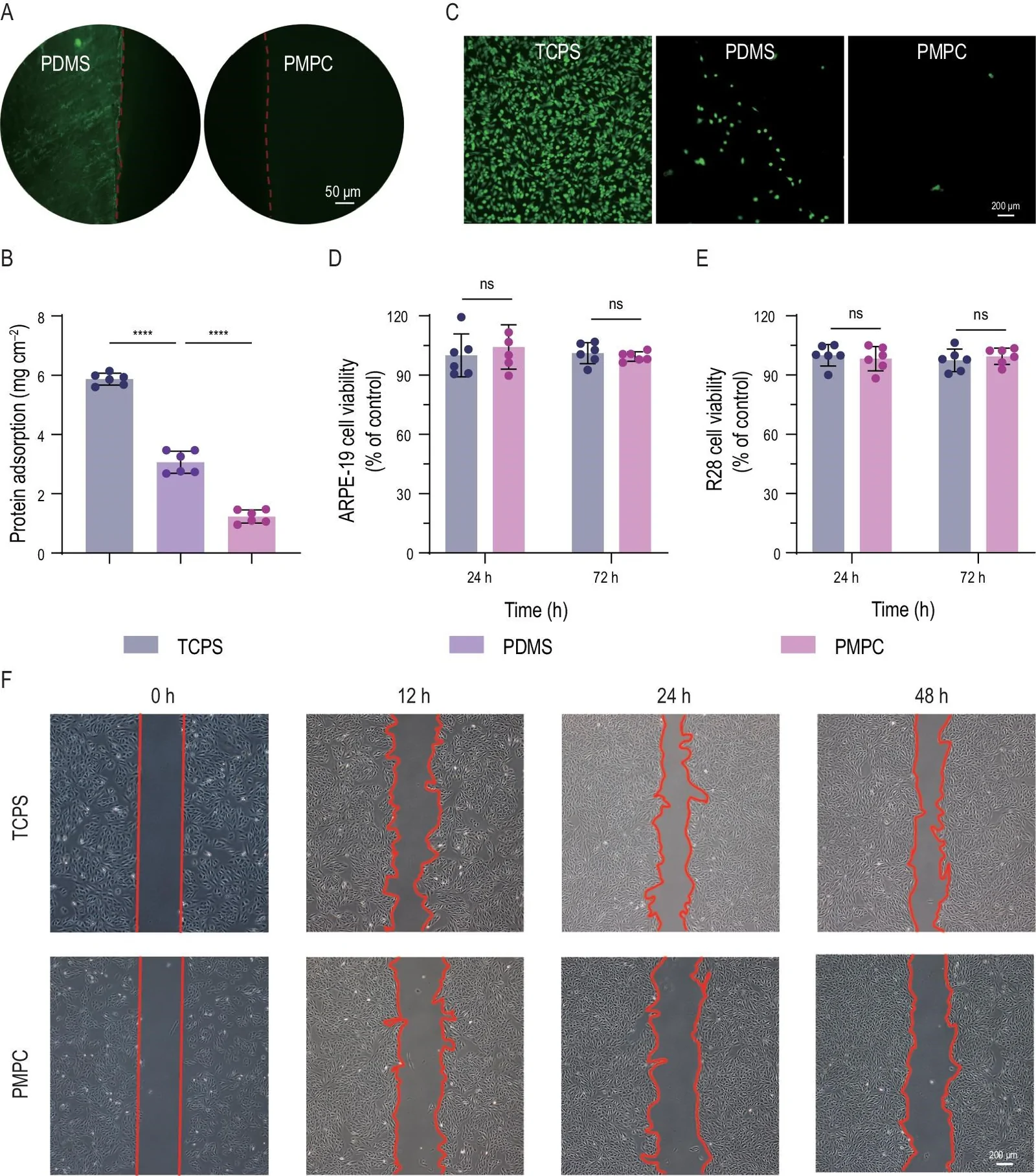
Antifouling capacity and *in vitro* biocompatibility of PMPC hydrogels. (A and B) Fluorescence microscopy images of FITC-BSA adsorption on PDMS and PMPC hydrogels (A) and the corresponding quantitative BSA adsorption on TCPS, PDMS and PMPC hydrogel surfaces (B) (scale bar, 50 μm). Data are presented as mean ± SD (*n* = 6). *P* values were calculated using one-way ANOVA followed by Tukey’s post hoc test. ****P < 0.0001. (C) Representative images of ARPE-19 cells attached to TCPS, PDMS and PMPC hydrogel surfaces (scale bar, 200 μm). (D and E) Relative viability of ARPE-19 cells (D) and R28 cells (E) after 24 h and 72 h co-culture with TCPS, PDMS and PMPC hydrogels. Data are presented as mean ± SD (*n* = 6). *P* values were calculated using an unpaired, two-tailed Student’s *t*-test. ns, not significant. (F) Representative migration images of ARPE-19 cells treated with TCPS and PMPC hydrogel for 0, 12, 24 and 48 h (scale bar, 200 μm).

Antifouling performance is essential to the long-term safety of vitreous substitutes. In the complex intraocular milieu, protein adsorption and subsequent cell adhesion to an implanted material can compromise optical clarity and exacerbate adverse tissue responses. We therefore systematically evaluated protein adsorption and cell responses on PMPC hydrogels. Protein adsorption was first assessed using fluorescein isothiocyanate-bovine serum albumin (FITC-BSA). Polydimethylsiloxane (PDMS) was included as a silicone-based surrogate to approximate the fouling propensity of SO. As shown in Fig. 2A, PDMS displayed strong green fluorescence, indicative of substantial protein adsorption, whereas PMPC hydrogels exhibited markedly lower fluorescence intensity, consistent with reduced non-specific protein deposition. Quantitative analysis (Fig. 2B) further showed that protein adsorption on PMPC hydrogel was 1.232 mg/cm^2^, substantially lower than that on tissue-culture polystyrene (TCPS, 5.868 mg/cm^2^) and PDMS (3.063 mg/cm^2^). Mechanistically, this antifouling behavior is attributed to the highly hydrated zwitterionic interface of PMPC, which suppresses non-specific protein deposition.

To assess cell–material interactions relevant to intraocular applications, we selected two retinal cell lines: human retinal pigment epithelial cells (ARPE-19), a widely used model of PVR-associated cellular responses, and retinal precursor cells (R28), a representative retinal cell type. We first evaluated the cytocompatibility of PMPC hydrogel toward both cell types. As shown in Fig. 2D and E and Fig. S6, the viability of ARPE-19 and R28 cells remained above 98% over 72 h for all PMPC groups, indicating negligible cytotoxicity and supporting further *in vivo* studies. We then examined cell adhesion on the hydrogel surfaces. Compared with TCPS and PDMS controls, both ARPE-19 and R28 exhibited minimal attachment and spreading on PMPC hydrogels (Fig. 2C and Fig. S7), consistent with lower non-specific protein adsorption. Given the central role of ARPE migration in PVR, we further assessed ARPE-19 migration using scratch assays, which revealed that PMPC hydrogel markedly suppressed ARPE-19 cell migration (Fig. 2F). This observation is particularly relevant because PVR is a common cause of vitrectomy failure, and ARPE-19 activation and migration are recognized as early cellular events that drive downstream proliferation, epithelial–mesenchymal transition and extracellular matrix deposition [37–40]. Therefore, the ability of PMPC hydrogel to restrain ARPE-19 migration, together with its low protein adsorption and anti-adhesive surfaces, suggests potential to mitigate early processes implicated in PVR progression.

### *In vivo* evaluation of PMPC hydrogels as vitreous substitutes

**Figure 3.**
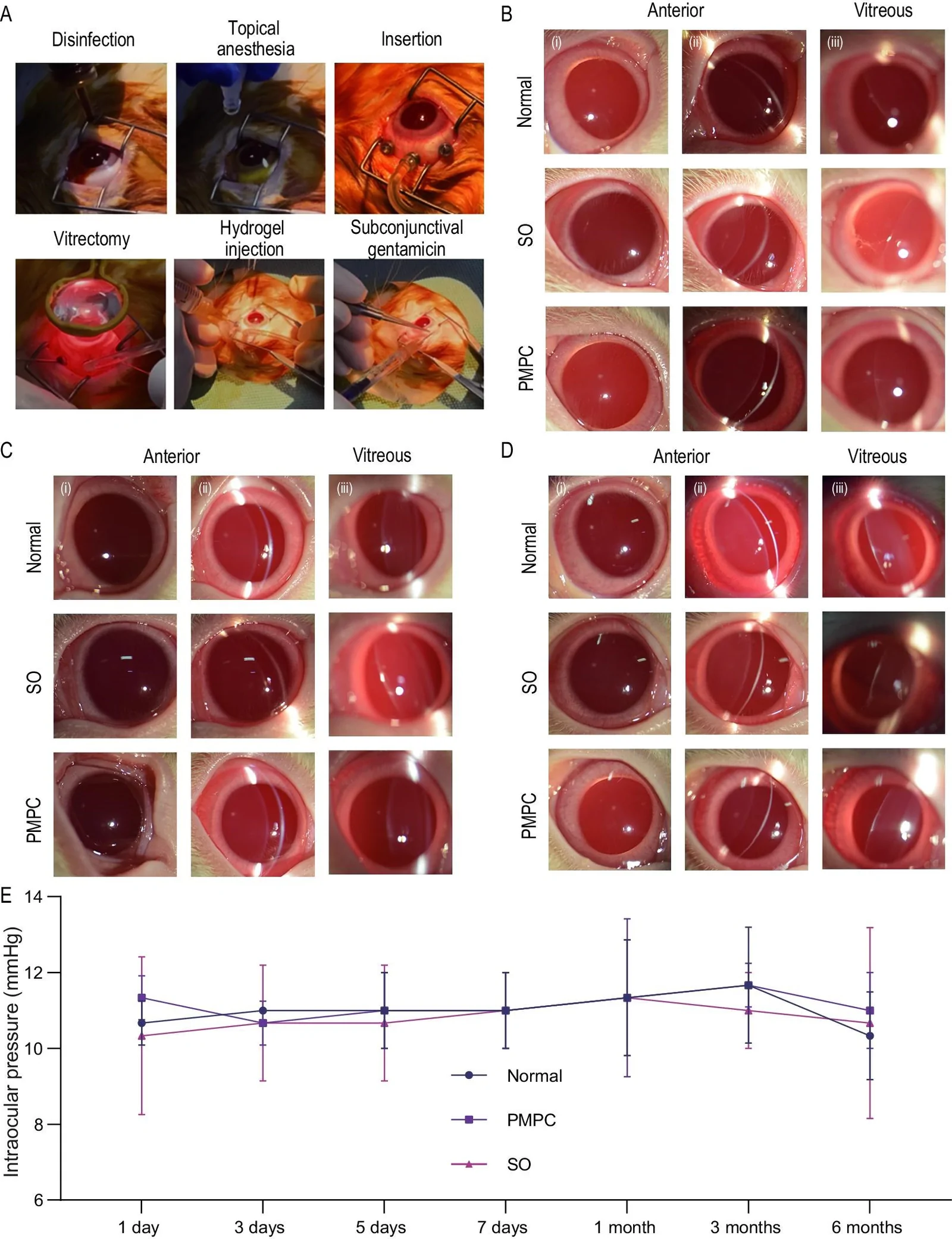
*In vivo* evaluation of PMPC hydrogels as vitreous substitutes. (A) Schematic of the standard vitrectomy performed under sterile conditions by a trained vitreoretinal surgeon, followed by intravitreal injection of the PMPC hydrogel into the vitrectomized eye. (B–D) Slit-Lamp images of Normal, SO and PMPC hydrogel groups at 1 month (B), 3 months (C) and 6 months (D) postoperatively. (i–iii) Images of the ocular surface (i), anterior chamber (ii) and vitreous cavity (iii). (E) IOP measured on postoperative days 1, 3, 5 and 7, as well as at 1, 3 and 6 months post implantation. Data are presented as mean ± SD (*n* = 3).

The *in vivo* ocular biocompatibility of the PMPC hydrogel was evaluated in New Zealand White rabbits following pars plana vitrectomy, as outlined in Fig. 3A. Briefly, the right eyes underwent 25-G vitrectomy and were then filled with either SO or the PMPC-35wt% hydrogel, while the contralateral left eyes were left untreated as controls (Video S1). Animals were followed for up to 6 months to monitor postoperative complications. Slit-lamp examinations (Fig. 3B–D) showed no signs of ocular irritation in the PMPC group throughout the 6-month observation period. The conjunctiva remained pale pink without apparent hyperemia or edema (Fig. 3B–D, i), and the anterior chamber stayed clear with no detectable aqueous flare (Fig. 3B–D, ii), suggesting an absence of anterior-segment inflammation. The vitreous cavity remained uniformly transparent with stable fundus reflexes and no visible opacities (Fig. 3B–D, iii). By comparison, the SO group exhibited enhanced interfacial reflections at 6 months (Fig. 3D, iii), which is likely attributable to optical mismatch and interface-related light scattering. Consistent with its vitreous-like refractive index (Table S1), the PMPC hydrogel is expected to preserve postoperative optical clarity better. IOP was monitored at predefined time points (Fig. 3E). IOP in the Normal, SO and PMPC groups remained within the physiological range throughout the study. No elevation suggestive of outflow obstruction was observed, and no decrease indicative of leakage-associated volume loss was detected. Together, these findings support the *in vivo* ocular safety of the PMPC hydrogel during long-term follow-up.

**Figure 4.**
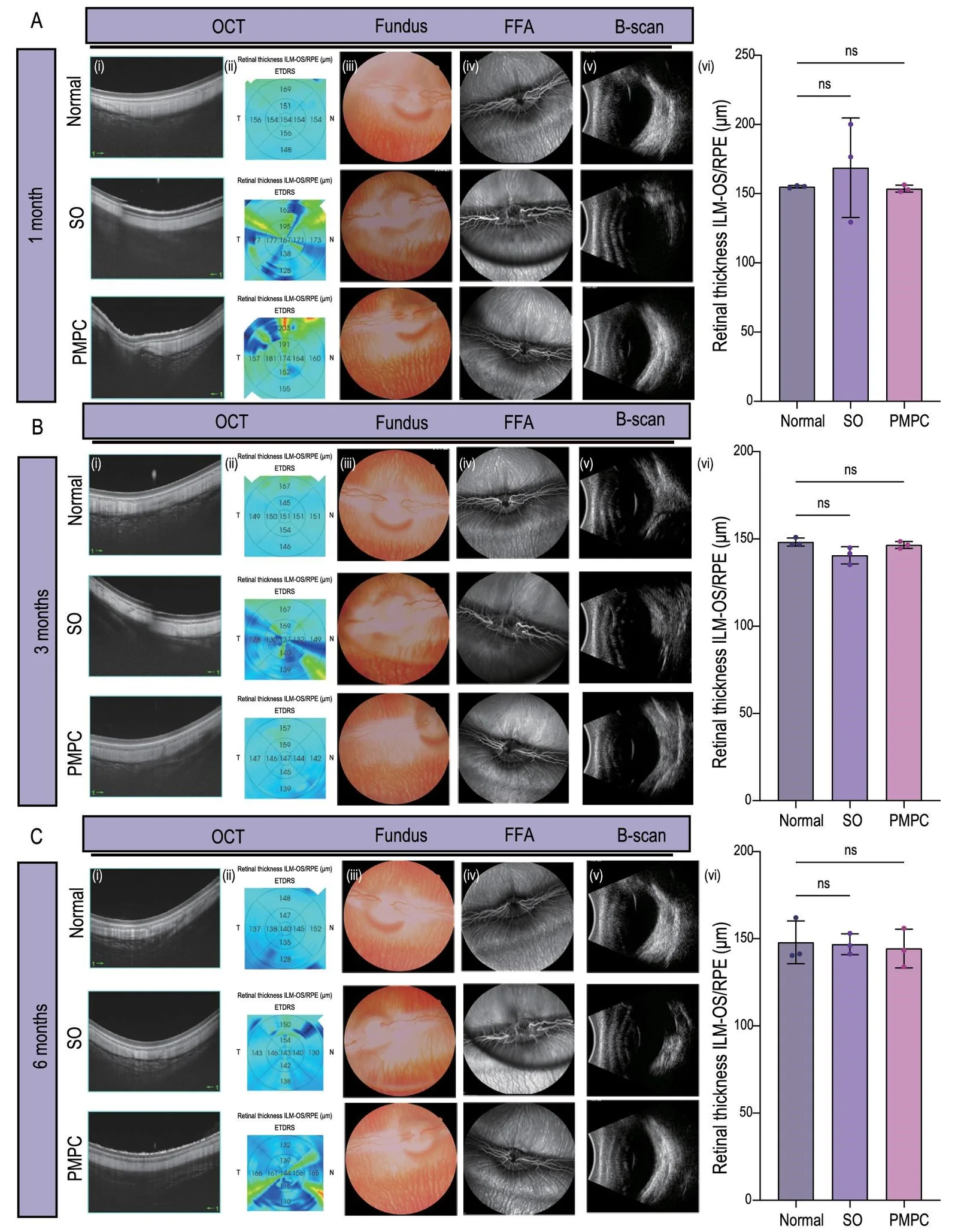
Representative images of OCT, fundus, FFA and B-scan. (A–C) Representative images of OCT, fundus, FFA and B-scan at 1 month (A), 3 months (B) and 6 months (C). (i–v) Representative retinal OCT images (i), retinal thickness map (ii), fundus images (iii), FFA images (iv) and B-scan ultrasonography images (v). (vi) Statistical analysis of retinal thickness. Data are presented as mean ± SD (*n* = 3). *P* values were calculated using one-way ANOVA followed by Tukey’s post hoc test. ns, not significant.

Retinal imaging was performed at predefined time points of 1, 3 and 6 months using optical coherence tomography (OCT) together with retinal thickness pseudocolor maps [Fig. 4A–C (i and ii)]. Across all groups and time points, OCT cross-sectional images showed well-defined retinal layer boundaries without apparent structural disruption, and the corresponding thickness maps remained spatially uniform within the imaged regions. Retinal thickness, defined as the distance between the inner limiting membrane and the outer segment/retinal pigment epithelium (OS/RPE) interface, showed no significant differences among the Normal, SO and PMPC groups at any time point [Fig. 4A–C (vi)]. These results suggest that PMPC hydrogel tamponade does not induce detectable thickness-related retinal abnormalities over 6 months, with no features consistent with retinal thinning or edema in the acquired scans. Fundus photography, fundus fluorescein angiography (FFA) and B-scan ultrasonography were employed to assess retinal morphology and vascular status. As shown in Fig. 4A–C (iii–v), the PMPC hydrogel group exhibited a normal orange-red fundus reflex with preserved vascular architecture and no obvious interface-related reflections throughout the 6-month follow-up. In contrast, the SO group showed localized enhancement of the fundus reflex, which is likely attributable to the refractive index mismatch between SO and the native vitreous, leading to interface-associated light scattering. During ocular rotation, the SO interface can shift, altering the optical path and producing dynamic reflections. Such optical instability is a plausible contributor to postoperative visual complaints, including image oscillation and distortion, reported with SO tamponade. In contrast, the refractive-index-matched PMPC hydrogel may minimize interface-related optical artifacts, potentially improving visual quality. FFA images [Fig. 4A–C (iv)] showed adequate vascular filling without obvious leakage, tortuosity or occlusion across all groups. In the SO group, mild localized hyperfluorescence was occasionally observed. This signal may arise from optical artifacts or subtle regional changes, and its underlying cause warrants further investigation. B-scan ultrasonography [Fig. 4A–C (v)] confirmed an attached retina without proliferative lesions in all groups. The PMPC hydrogel filled the vitreous cavity without obvious interface separation.

We subsequently employed electroretinography (ERG) to assess retinal function under both scotopic (dark-adapted, DA) and photopic (light-adapted, LA) conditions. In principle, the ERG a-wave and b-wave primarily reflect the functional integrity of photoreceptors and the ON-bipolar/Müller cell pathway, respectively. The DA 0.01, DA 3.0 and LA 3.0 responses represent the rod-specific response, the combined rod–cone maximal response and the cone-mediated response, respectively, where 0.01 and 3.0 correspond to stimulus intensities of 0.01 and 2.562 cd s/m^2^. Representative ERG waveforms are presented in Figs S8 and S9. At 1 month post implantation, the DA 0.01 b-wave in the PMPC hydrogel group showed a slight reduction (Fig. S10A), which may reflect transient functional changes associated with surgical manipulation. By 3 months, the response substantially recovered. As shown in Fig. S10B and C, no significant differences were observed in the a-wave or b-wave amplitudes under DA 3.0 between the PMPC hydrogel group and the Normal group. The amplitudes of the LA 3.0 b-wave remained comparable throughout follow-up (Fig. S10D), indicating preserved cone-mediated retinal function. Collectively, these results suggest that long-term intraocular presence of the PMPC hydrogel does not cause detectable impairment in retinal electrophysiological function.

### Rhegmatogenous retinal detachment model

**Figure 5.**
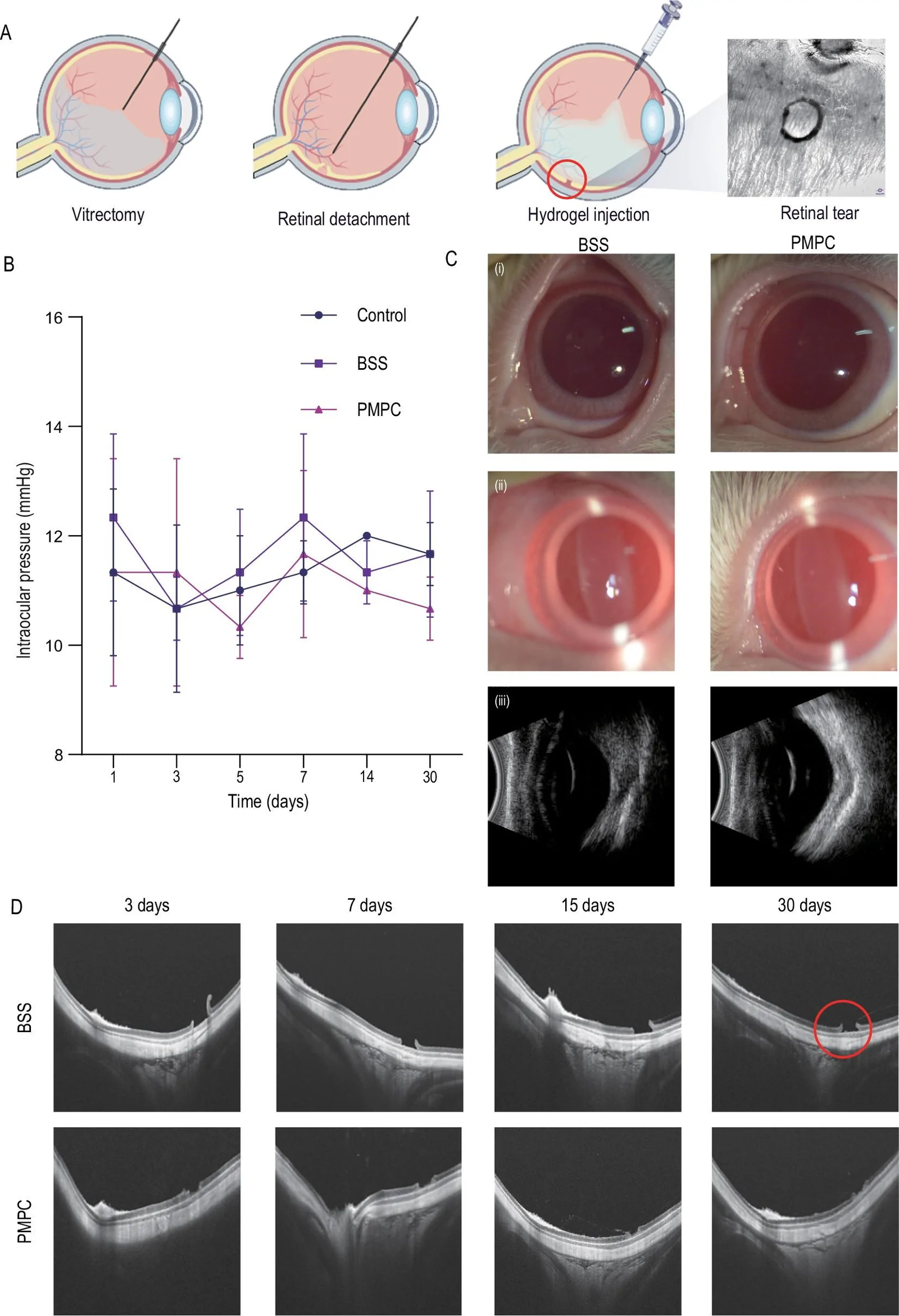
Rhegmatogenous RD model. (A) Schematic illustration of the surgical procedure used to produce vitrectomy, RD and hydrogel injection in the rabbit model. (B) IOP was measured on postoperative days 1, 3, 5, 7, 14 and 30. (C) Slit-lamp images and B-scan ultrasonography images at 1 month postoperatively. (i–iii) Images of the ocular surface (i), the vitreous cavity (ii) and B-scan ultrasonography (iii). (D) Representative OCT images showing retinal status after surgery at days 3, 7, 15 and 30. The red circle exhibits recurrent RD adjacent to the break. Data are presented as mean ± SD (*n* = 3).

Building on the long-term ocular tolerance observed in the standard post-vitrectomy replacement model, we further assessed the tamponade function of the PMPC hydrogel in a more stringent rhegmatogenous RD model (Fig. 5A). In this exploratory study, PMPC hydrogel was compared with balanced salt solution (BSS) after creation of a retinal break. IOP remained within the physiological range throughout the 30-day follow-up (Fig. 5B). Slit-lamp examinations showed a quiet anterior segment in the PMPC group without obvious inflammatory signs or cataract formation, and B-scan ultrasonography revealed no abnormal findings suggestive of proliferative lesions (Fig. 5C). Representative longitudinal OCT images from a successfully treated PMPC eye showed sustained break closure and retinal reattachment from day 3 to 1 month (Fig. 5D). In contrast, BSS-treated eyes exhibited recurrent detachment adjacent to the break by 1 month (Fig. 5D, red circle). Notably, re-detachment was also observed in some PMPC-treated eyes within the 30-day period, indicating that while the feasibility of tamponade using PMPC hydrogel can be demonstrated in this disease-relevant model, further optimization and expanded validation are warranted.

### Histological evaluation of PMPC hydrogels as vitreous substitutes

At 6 months post implantation, the PMPC hydrogel could be retrieved intact with forceps, indicating stable residence in the vitreous cavity and preservation of its macroscopic integrity (Fig. S11A). Histological assessments were then performed to further examine ocular tissue compatibility. Hematoxylin and eosin (H&E) staining of the anterior segment, including the cornea, iris and ciliary body, showed well-preserved tissue morphology in the PMPC group, with no overt signs of vascular congestion, edema or inflammatory cell infiltration compared with the Normal and SO groups [Fig. S11B (i–iii)]. Retinal H&E sections further revealed intact lamination with clearly defined boundaries across major layers, including the nerve fiber layer, ganglion cell layer, inner plexiform layer, inner nuclear layer (INL), outer plexiform layer (OPL) and outer nuclear layer (ONL) (Fig. S11C(i)). Consistently, Masson’s trichrome staining showed preserved retinal architecture without apparent fibrotic remodeling in the PMPC group at 6 months [Fig. S11C (ii)]. We next evaluated glial reactivity by immunostaining for glial fibrillary acidic protein (GFAP), a marker of reactive gliosis primarily associated with Müller cell activation. GFAP signals were only mildly increased in both the SO and PMPC groups and were mainly confined to inner retinal regions [Fig. S11C(iii)], which likely reflects a limited response to surgical manipulation rather than material-driven chronic irritation. To further quantify retinal glial activation, integrated optical density (IOD) analysis of GFAP immunostaining was performed. No significant difference in GFAP IOD was observed among the Normal, SO and PMPC groups (Fig. S12), further indicating that long-term PMPC hydrogel implantation did not induce obvious GFAP upregulation or retinal gliosis. At 6 months, we performed H&E and Masson’s trichrome staining of major organs, including the heart, liver, spleen, lungs and kidneys, in the rabbits. As shown in Fig. S13, the long-term implantation of the PMPC hydrogel did not induce significant systemic toxicity or elicit systemic fibrotic responses. These results demonstrate the favorable biosafety profile of the material at the systemic level.

## CONCLUSION

In summary, we present a minimalist injectable hydrogel design for vitreous replacement in which a macroscopic PMPC network forms without external crosslinkers or engineered associative motifs, with mechanical integrity primarily governed by topological chain entanglements. The resulting PMPC hydrogel exhibits physicochemical properties suitable for vitreous replacement (e.g., density of 1.011 g/cm^3^ and surface tension of 39.65 mN/m) and reproduces the optical and viscoelastic characteristics of the native vitreous. Owing to its highly hydrated interface, the hydrogel exhibits strong antifouling performance, effectively suppressing protein adsorption and bacterial adhesion, and further attenuating ARPE-19 cell migration, thereby potentially interfering with early adhesion-to-migration events associated with PVR progression. Importantly, in a rabbit vitrectomy model with longitudinal follow-up to 6 months, multimodal ophthalmic examinations, including slit-lamp imaging, OCT, B-scan ultrasonography, fundus photography, FFA and ERG, collectively support the long-term intraocular safety and functional stability of the PMPC hydrogel. Building on these findings, we further assessed tamponade-relevant performance in a rabbit rhegmatogenous RD model, where the hydrogel served as an internal tamponade to appose the detached neurosensory retina to the underlying RPE, thereby supporting break coverage and sustained retinal reattachment over the observation period. Taken together, this work couples an entanglement-stabilized, crosslinker-free network with a zwitterionic polymer building block that has established industrial availability and regulatory/clinical precedent, offering a feasible materials path toward a practical SO alternative while reducing formulation complexity and translational burden.

## MATERIALS AND METHODS

Detailed materials and methods can be found in the online Supplementary data.

### Ethical statements

All the animal experiments complied with the guidelines of the Tianjin Medical Experimental Animal Care, and animal protocols were approved by the Institutional Animal Care and Use Committee of Yi Shengyuan Gene Technology (Tianjin) Co., Ltd. (protocol number YSY-DWLL-2025 859).

## Supplementary Material

nwag413_Supplemental_Files

## References

[1] Kleinberg TT, Tzekov RT, Stein L et al. Vitreous substitutes: a comprehensive review. Surv Ophthalmol 2011; 56: 300–23. 10.1016/j.survophthal.2010.09.001 21601902

[2] Ribeiro L, Oliveira J, Kuroiwa D et al. Advances in vitreoretinal surgery. J Clin Med 2022; 11: 6428. 10.3390/jcm11216428 36362657 PMC9658321

[3] Schulz A, Keskar M, Swindle-Reilly KE et al. Replacing the vitreous body with hydrogels: rationale and strategies. Prog Retin Eye Res 2025; 108: 101389. 10.1016/j.preteyeres.2025.101389 40645475 PMC13134038

[4] Forman A, Baker AEG, Shoichet MS. Vitreous substitutes. In: Wells L, Sheardown H (eds). Ophthalmic Biomaterials. Cambridge: Royal Society of Chemistry, 2025, 72–96. 10.1039/9781839169779

[5] Gao Q-Y, Fu Y, Hui Y-N. Vitreous substitutes: challenges and directions. Int J Ophthalmol 2015; 8: 437–40. 10.3980/j.issn.2222-3959.2015.03.01 26085987 PMC4458642

[6] Su X, Tan MJ, Li Z et al. Recent progress in using biomaterials as vitreous substitutes. Biomacromolecules 2015; 16: 3093–102. 10.1021/acs.biomac.5b01091 26366887

[7] Chen Y, Kearns VR, Zhou L et al. Silicone oil in vitreoretinal surgery: indications, complications, new developments and alternative long-term tamponade agents. Acta Ophthalmol 2021; 99: 240–50. 10.1111/aos.14604 32930501

[8] Lin Q, Lim JYC, Xue K et al. Polymeric hydrogels as a vitreous replacement strategy in the eye. Biomaterials 2021; 268: 120547. 10.1016/j.biomaterials.2020.120547 33307366

[9] Gong Q, Zhao Y, Qian T et al. Functionalized hydrogels in ophthalmic applications: ocular inflammation, corneal injuries, vitreous substitutes and intravitreal injection. Mater Des 2022; 224: 111277. 10.1016/j.matdes.2022.111277

[10] Nguyen M, Karkanitsa M, Christman KL. Design and translation of injectable biomaterials. Nat Rev Bioeng 2024; 2: 810–28. 10.1038/s44222-024-00213-1

[11] Sojdeh S, Panjipour A, Bejandi ZB et al. Hydrogel-based vitreous substitutes. Int J Mol Sci 2025; 26: 8406. 10.3390/ijms26178406 40943326 PMC12428293

[12] Zhang L, Cao Z, Bai T et al. Zwitterionic hydrogels implanted in mice resist the foreign-body reaction. Nat Biotechnol 2013; 31: 553–7. 10.1038/nbt.2580 23666011

[13] Li Q, Wen C, Yang J et al. Zwitterionic biomaterials. Chem Rev 2022; 122: 17073–154. 10.1021/acs.chemrev.2c00344 36201481

[14] Pang Y, Wang H, Yao Y et al. An injectable self-crosslinked wholly supramolecular polyzwitterionic hydrogel for regulating microenvironment to boost infected diabetic wound healing. Adv Funct Mater 2023; 33: 2303095. 10.1002/adfm.202303095

[15] Wang Z, Chen D, Wang H et al. The unprecedented biodegradable polyzwitterion: a removal-free patch for accelerating infected diabetic wound healing. Adv Mater 2024; 36: 2404297. 10.1002/adma.202404297 38734972

[16] Chang J, Tao Y, Wang B et al. An in situ-forming zwitterionic hydrogel as vitreous substitute. J Mater Chem B 2015; 3: 1097–105. 10.1039/C4TB01775G 32261988

[17] Wang H, Wu Y, Cui C et al. Antifouling super water absorbent supramolecular polymer hydrogel as an artificial vitreous body. Adv Sci 2018; 5: 1800711. 10.1002/advs.201800711 PMC6247043 30479921

[18] He B, Yang J, Liu Y et al. An in situ-forming polyzwitterion hydrogel: towards vitreous substitute application. Bioact Mater 2021; 6: 3085–96. 10.1016/j.bioactmat.2021.02.029 33778190 PMC7960944

[19] Cai Y, Tan Y, Cao J et al. Dual-crosslinked betaine-based amphiphilic hydrogel as a promising vitreous substitute: anti-adhesion, anti-fouling, and anti-cell proliferation. Adv Sci 2025; 12: e13455. 10.1002/advs.202413455 PMC12462983 40586226

[20] Lang L, Hao H, Yao J et al. Purely zwitterionic polymer injectable hydrogels for vitreous substitutes. Sci China Mater 2025; 68: 3390–400. 10.1007/s40843-025-3620-x

[21] Ishihara K . Blood-compatible surfaces with phosphorylcholine-based polymers for cardiovascular medical devices. Langmuir 2019; 35: 1778–87. 10.1021/acs.langmuir.8b01565 30056709

[22] Ishihara K . Biomimetic materials based on zwitterionic polymers toward human-friendly medical devices. Sci Technol Adv Mater 2022; 23: 498–524. 10.1080/14686996.2022.2119883 36117516 PMC9481090

[23] Ishihara K . Biomimetic polymers with phosphorylcholine groups as biomaterials for medical devices. Proc Jpn Acad Ser B Phys Biol Sci 2024; 100: 579–606. 10.2183/pjab.100.037 PMC11704457 39662944

[24] Goda T, Ishihara K. Soft contact lens biomaterials from bioinspired phospholipid polymers. Expert Rev Med Devices 2006; 3: 167–74. 10.1586/17434440.3.2.167 16515383

[25] Ishihara K . Revolutionary advances in 2-methacryloyloxyethyl phosphorylcholine polymers as biomaterials. J Biomed Mater Res A 2019; 107: 933–43. 10.1002/jbm.a.36635 30701666

[26] Wang Z, Li J, Liu Y et al. Synthesis and characterizations of zwitterionic copolymer hydrogels with excellent lubrication behavior. Tribol Int 2020; 143: 106026. 10.1016/j.triboint.2019.106026

[27] Nguyen HN, Ngo TLH, Iwasaki Y et al. Biodegradable phosphocholine cross-linker with ion-pair design for tough zwitterionic hydrogel. Adv Mater Interfaces 2022; 9: 202201002. 10.1002/admi.202201002

[28] He K, Cai P, Ji S et al. An antidehydration hydrogel based on zwitterionic oligomers for bioelectronic interfacing. Adv Mater 2024; 36: 2311255. 10.1002/adma.202311255 38030137

[29] Zhou B, Li Y, Chen Y et al. Initiator-free supramolecular zwitterionic gels for skin-inspired soft iontronics. Adv Mater 2026; 38: e14037. 10.1002/adma.202514037 41388507

[30] Kim J, Zhang G, Shi M et al. Fracture, fatigue, and friction of polymers in which entanglements greatly outnumber cross-links. Science 2021; 374: 212–6. 10.1126/science.abg6320 34618571

[31] Nian G, Kim J, Bao X et al. Making highly elastic and tough hydrogels from doughs. Adv Mater 2022; 34: 2206577. 10.1002/adma.202206577 36126085

[32] Wang H, Liu B, Chen D et al. Low hysteresis zwitterionic supramolecular polymer ion-conductive elastomers with anti-freezing properties, high stretchability, and self-adhesion for flexible electronic devices. Mater Horiz 2024; 11: 2628–42. 10.1039/D4MH00174E 38501271

[33] Kang T, Dai J, Huang Y et al. Entanglements and fracture in polymers. Chem Rev 2025; 125: 11032–57. 10.1021/acs.chemrev.5c00459 41043043

[34] Song X, Man J, Wang X et al. Pure zwitterionic hydrogels with high entanglement reinforcement for biomedical applications. ACS Appl Mater Interfaces 2025; 17: 48094–110. 10.1021/acsami.5c12081 40826988

[35] Wei A, Wang Q, Liu J et al. Co-initiating-system dual-mechanism drives the design of printable entangled polymer multinetworks. Nat Commun 2025; 16: 4407. 10.1038/s41467-025-59669-3 40355471 PMC12069718

[36] Cao W, Zhou X, Dai W et al. An embolism-free nonfouling hydrogel coating with high toughness and lubricity for intravascular medical devices via chain-entanglement mediated topological gelation. Bioact Mater 2026; 59: 174–85. 10.1016/j.bioactmat.2025.11.033 41536917 PMC12796771

[37] He S, Kumar SR, Zhou P et al. Soluble EphB4 inhibition of PDGF-induced RPE migration in vitro. Invest Ophthalmol Visual Sci 2010; 51: 543–52. 10.1167/iovs.09-3475 19696168 PMC2828363

[38] Hou Q, Zhou L, Tang J et al. LGR4 is a direct target of microRNA-34a and modulates the proliferation and migration of retinal pigment epithelial ARPE-19 cells. PLoS One 2016; 11: e0168320. 10.1371/journal.pone.0168320 27977785 PMC5158047

[39] Ko JA, Sotani Y, Ibrahim DG et al. Role of macrophage migration inhibitory factor (MIF) in the effects of oxidative stress on human retinal pigment epithelial cells. Cell Biochem Funct 2017; 35: 426–32. 10.1002/cbf.3292 28906008

[40] Huang Y, Meister R, Lindziute M et al. A TGFB_2_/TNF-induced in vitro model of proliferative vitreoretinopathy (PVR) using ARPE-19 cells confirms nicotinamide as an inhibitor of EMT and VEGFA secretion. PLoS One 2026; 21: e0340614. 10.1371/journal.pone.0340614 41529022 PMC12798965

