# Supplementary material for: Chain entanglement-stabilized injectable zwitterionic hydrogel for vitreous replacement and retinal tamponade
Source: Natl Sci Rev. 2026 Jul 6;13(17):nwag413. doi: 10.1093/nsr/nwag413 (PMC13576433; doi:10.1093/nsr/nwag413)
Supplement: Supplementary file 1 [file nwag413_supplemental_files.zip › Supplementary data.pdf]

Supplemental information

Chain entanglement-stabilized injectable zwitterionic hydrogel for vitreous replacement and retinal tamponade

Haolun Wang[1,†], Hongying Wang[1,†], Liping Lang[1,†], Huijie Hao[1,2], Yuqin Cheng[1], Danyang Chen[1], Xu Tian[1], Boshi Liu[2], Xiaoli Xing[2,*], Xiaorong Li[2,*], Wenguang Liu[1,*], and Jianhai Yang[1,*]

[1]School of Materials Science and Engineering, State Key Laboratory of Precious Metal Functional Materials, Tianjin University, Tianjin 300350, China

[2]Tianjin Key Laboratory of Retinal Functions and Diseases, Tianjin Branch of National Clinical Research Center for Ocular Disease, Eye Institute and School of Optometry, Tianjin Medical University Eye Hospital, Tianjin 300384, China

[†]Equally contributed to this work.

***Corresponding authors**. E-mails: jianhaiyang@tju.edu.cn; wgliu@tju.edu.cn; lixiaorong@tmu.edu.cn; xxlteh@126.com.

METHODS

Materials

2-methacryloyloxyethyl phosphorylcholine (MPC) was purchased from Bide Pharmatech Co., Ltd. 2-Hydroxy-2-methylpropiophenone (IRGACURE 1173, 97%) was purchased from Sigma-Aldrich (Shanghai, China). CCK-8 was obtained from Beyotime Biotechnology (Shanghai, China). Silicone oil (SO, S5.7570) was obtained from French Surgical Instruments SAS. All other chemicals and solvents were of analytical grade and were used as received without any further purification.

Preparation of poly(2-methacryloyloxyethyl phosphorylcholine) (PMPC) hydrogels

A specified amount of MPC was dissolved in deionized water to form a homogeneous solution, to which the photoinitiator IRGACURE 1173 (0.1 mol% relative to MPC) was added. The resulting solution was poured into a custom mold and placed in a UV crosslinking chamber (XL-1000 UV crosslinker, Spectronics Corporation, New York, USA), where it was irradiated for one hour to form PMPC hydrogels. For simplicity, the resulting hydrogels are denoted as PMPC-X, where X represents the initial MPC concentration (wt%). For *in vitro* and *in vivo* studies, hydrogels were prepared under aseptic conditions. Deionized water was autoclaved, and all other reagents were sterile filtered before use. The entire process was performed aseptically using sterilized glassware and filtration components in a biological safety cabinet.

Mechanical tests

Tensile testing was conducted at room temperature using a universal testing machine (Instron 2344, Instron, USA) equipped with a 500 N load cell. Freshly prepared hydrogel samples were cut into dumbbell-shaped specimens and stretched at a constant rate of 50 mm/min. Stress was calculated by dividing the applied force by the specimen’s cross-sectional area, while strain was defined as the ratio of the extension to the original gauge length. The Young’s modulus was determined from the slope of the linear region (10–20% strain) of the stress–strain curve. Toughness was calculated

as the area under the corresponding stress–strain curve. For each hydrogel prepared at a different monomer concentration, five independent specimens were tested, and results are presented as mean ± standard deviation.

Characterizations

Refractive indices were measured using an Abbe refractometer (WYA-2W, Lumsail Industrial Inc., Shanghai, China). Measurements were performed under controlled temperature conditions required by the experimental protocol. Optical transmittance of PMPC hydrogels was measured using a UV-visible spectrophotometer (GENESYS 180, Thermo Fisher) across a wavelength range of 300–800 nm, with phosphate-buffered saline (PBS) as the reference. Surface morphology of PMPC hydrogels was observed using a scanning electron microscope (SEM, Quattro S, Thermo Fisher Scientific, USA) operated at 10 kV. For SEM preparation, hydrogel samples were quickly frozen in liquid nitrogen to crystallize the water within the hydrogel, then lyophilized for at least 24 h to sublimate the ice crystals. The freeze-dried samples were placed on a plate filled with liquid nitrogen, cut with a very fine blade to expose the cross-section, and finally sprayed with gold.

Surface tension measurements

The surface tension of PMPC hydrogels was measured using the hanging drop method with a contact angle/surface tension analyzer (OCA20, Dataphysics, German). A droplet was gently dispensed onto the hydrogel surface, and its shape was recorded via the instrument's integrated microscopy system. The contact angle and droplet radius were obtained from the captured images, and surface tension was subsequently calculated using standard geometric analysis.

Permeability measurements

PMPC hydrogel sheets were placed at the bottom of a glass vials, followed by the addition of 10 mL of sodium fluorescein solution in PBS. Images were captured at specific time intervals: 1, 5, 10, and 60 minutes, as well as 6 and 24 hours. To further evaluate hydrogel permeability, the sodium fluorescein solution was removed after 24 hours of contact with the PMPC hydrogel and replaced with 10 mL of fresh PBS.

Images were then taken at the same intervals: 1, 5, 10, and 60 minutes, followed by 6 and 24 hours. The PBS was replaced with fresh solution after each imaging time point. For comparative analysis, 200 μL of SO and 200 μL of PMPC hydrogel were extruded through a 25-G needle and placed at the bottom of glass vials. Subsequently, 1 mL of PBS-diluted sodium fluorescein solution was added to each vial, and images were recorded at intervals of 1, 5, and 60 minutes.

Rheological analysis

The rheological properties of PMPC hydrogels were characterized using a rheometer (MCR302, Anton Paar) equipped with a 25 mm diameter cone plate, with all measurements conducted at 37 °C. To determine the linear viscoelastic region (LVR), oscillatory strain sweeps were performed from 0.1% to 1000% at a constant frequency of 1 Hz. A time-sweep test was subsequently carried out at 1 Hz frequency and 0.1% strain for 180 s to evaluate the temporal stability of the hydrogel network. Frequency-dependent behavior was assessed by performing frequency sweeps from 0.01 to 10 Hz at a fixed strain of 0.1%, with both storage modulus (G′) and loss modulus (G″) recorded. To investigate shear-thinning behavior, viscosity was measured across a shear rate ranging from 0.001 to 1000 1/s. For self-healing and recovery analysis, alternating strains of 1% and 1500% were applied in a stepwise fashion. Shear creep tests were conducted under constant stresses of 0.5 Pa and 1.0 Pa, with each phase followed by 100 s of creep and recovery. Creep compliance and strain evolution were continuously monitored throughout the test.

Measurement of nonspecific protein adsorption

Protein adsorption on PMPC hydrogels and polydimethylsiloxane (PDMS) was evaluated using a fluorescence-based assay. Briefly, hydrogel sheets (6 mm × 6 mm) were incubated at room temperature in a 0.1 mg/mL solution of fluorescein Isothiocyanate-bovine serum albumin (FITC-BSA) for 30 minutes. After incubation, samples were gently rinsed three times with PBS to remove loosely bound proteins. For quantitative analysis, a separate set of equilibrated hydrogel sheets was immersed in 1 mL of BSA solution (2 mg/mL) and incubated at 37 °C for 90 minutes. After rinsing

three times with PBS, the absorbed proteins were desorbed via ultrasonication. The concentration of the adsorbed proteins was determined using a micro-BCA protein assay kit (Boster Biological Technology Co., Ltd.) according to the manufacturer's protocol. The amount of adsorbed protein was calculated based on a standard curve prepared with known BCA concentrations. All experiments were performed in triplicate with independent samples. For comparison of antifouling performance, SO was substituted with PDMS elastomer sheets due to the impracticality of direct testing with liquid SO. The amount of nonspecific protein adsorption on the PDMS elastomer was quantified following the same experimental procedure.

Cell attachment and cytotoxicity assay

ARPE-19 and R28 cells were used to assess the cell attachment of PMPC hydrogels. Fully swollen, sterile hydrogel sheets were placed at the bottom of a 24-well plate. R28 and ARPE-19 cells were then seeded at a density of 1×10^5 cells/well. After 48 hours of incubation at 37 °C in a 5% CO_2 atmosphere, the medium was removed, and the hydrogels were gently rinsed with PBS to remove any suspended cells. Cell adhesion was observed using an inverted microscope. To assess morphology, live and dead cell staining was performed using a calcein-AM and propidium iodide (PI) mix, incubating for 10 minutes at 37 °C in the dark. Excess dye was washed off with sterile PBS, and images were captured under the inverted microscope. Live cells were stained green, and dead cells were stained red, with tissue-culture polystyrene (TCPS) and PDMS used as controls for comparison.

PMPC hydrogels were co-cultured with R28 or ARPE-19 cells to evaluate their cytocompatibility using a CCK-8 assay kit. R28 and ARPE-19 cells were seeded in a 96-well plate at a density of 1×10^4 cells/well, with 200 μL of complete medium added to each well. The cells were incubated at 37 °C in a 5% CO_2 atmosphere for 24 hours and 72 hours. Following this, the original medium was aspirated, and 200 μL of PMPC hydrogel extract was added to each well. The group of cells incubated with a complete medium served as the control. After 24 hours of culture, the liquid in each well was aspirated, and the plate was gently rinsed with PBS. Then, 100 μL of complete medium

and 10 μL of CCK-8 reagent were added to each well and incubated for 3 hours at 37 °C in a 5% CO_2 atmosphere, and absorbance at 450 nm was measured using a microplate reader (Infinite M200 PRO, Tecan, Switzerland). Relative cell viability was calculated using the following formula:

$$Cell\ viability = \frac{Abs_{sample}}{Abs_{control}} \times 100\% \qquad \text{(Equation 1)}$$

Among them, Abs_{sample} is the absorbance of the experimental group, and$Abs_{control}$ is the absorbance of the control group.

In vitro cell wound scratch assay. ARPE-19 and R28 cells were seeded into 24-well plates at a density of 8×10^4 cells/well. Once the cells had successfully adhered and reached 100% confluence, a 10-μL pipette tip was used to create straight scratches on the surface of the wells. After washing off the non-adherent cells, culture medium containing hydrogel extract was added to the wells, and the cells were further cultured. Images were captured at 0, 12, 24, and 48 hours to observe the effect of the hydrogel on the migration of ARPE-19 and R28 cells.

Vitreous substitution surgery

All the animal experiments complied with the guidelines of the Tianjin Medical Experimental Animal Care, and animal protocols were approved by the Institutional Animal Care and Use Committee of Yi Shengyuan Gene Technology (Tianjin) Co., Ltd. (protocol number YSY-DWLL-2025859). Before surgery, 20 New Zealand White rabbits were acclimated to the laboratory environment for one week. General anesthesia was induced by intramuscular injection of Zota 50 (0.1 mL) and xylazine (0.3 mL). Tropicamide compound eye drops were administered for pupil dilation, and lidocaine hydrochloride eye drops were used for topical anesthesia. BSS was used as the infusion fluid. Posterior vitreous detachment was created. Vitrectomy was performed on the right eyes of all rabbits using a 25-G vitrectomy system without the use of triamcinolone acetonide for staining. Following vitreous removal, the replacement material was injected into the vitreous cavity using a 25-G syringe needle. Among the experimental animals, 5 rabbits received PMPC hydrogels, and 5 were administered

SO; the untreated left eyes served as internal controls. For the retinal detachment model, after removal of the vitreous body, a retinal tear approximately 2–3 disc diameters was created 1.5–2 μm below the optic disc using a backflush soft tip (Alcon), and BSS was injected through the tear site to induce retinal detachment. After retinal detachment, fluid-air exchange was performed through the break site to achieve temporary retinal reattachment and visually confirm the attached retina, 5 rabbits received PMPC hydrogels, and 5 were administered BSS. Following implantation, all scleral incisions were closed with 8-0 sutures and surgeries were consistently performed by a single experienced surgeon to reduce procedural variability. For postoperative care, gentamicin was administered via subconjunctival injection immediately after surgery, and tobramycin-dexamethasone ophthalmic ointment was applied twice daily for one week to prevent infection.

Postoperative ocular examination

Intraocular pressure (IOP) was assessed using a rebound tonometer at 6 months post-operation. B-scan ultrasonography, fundus photography, and fluorescein angiography were conducted for 1 month following vitreous replacement. For angiographic imaging, 0.2 mL of fluorescein sodium was intravenously injected into the rabbit's marginal ear vein to enhance visualization of retinal vasculature. Slit-lamp was applied to examine the leakage of hydrogel from the sclera puncture and the inflammation of the anterior segment. Use 5% tropicamide for pupil dilation and anesthetize the animals. Collect the retinal electrograph (ERG) data under bright adaptation, then expose the animals to dark adaptation for 30 minutes. The various components of the ERG examination are named according to the adaptation status of the eyes (dark adaptation (DA); bright adaptation (LA)) and the stimulus intensity per square meter per second (candela seconds). Here, DA 0.01 indicates the use of 0.01 candela seconds per square meter of light flash in the dark adaptation state, while LA 3 indicates the use of 2.562 candela seconds per square meter of light flash in the bright vision condition. At six months post-operation, the animals were euthanized, and both the right (operated) and left (control) eyes were enucleated. The eyes were immediately fixed in a specialized ocular

fixative solution, then embedded in paraffin. Sections were prepared and stained with Hematoxylin and Eosin (H&E) and Masson's trichrome for histological evaluation. The stained sections were visualized and analyzed under a microscope. Concurrently, major organs—including the heart, liver, spleen, lungs, and kidneys—were collected and processed for H&E staining to assess systemic biocompatibility.

Statistical analysis

Statistical analysis was performed using GraphPad Prism 10. Data are presented as mean ± standard deviation (SD). Comparisons between two groups were conducted by an unpaired, two-tailed Student's t test. Comparison of multiple groups was analyzed by one-way analysis of variance (ANOVA) followed by Tukey's post hoc test. Statistical significance was defined as $*p < 0.05$, $**p < 0.01$, $***p < 0.001$, and $****p < 0.0001$.

Supplementary figures

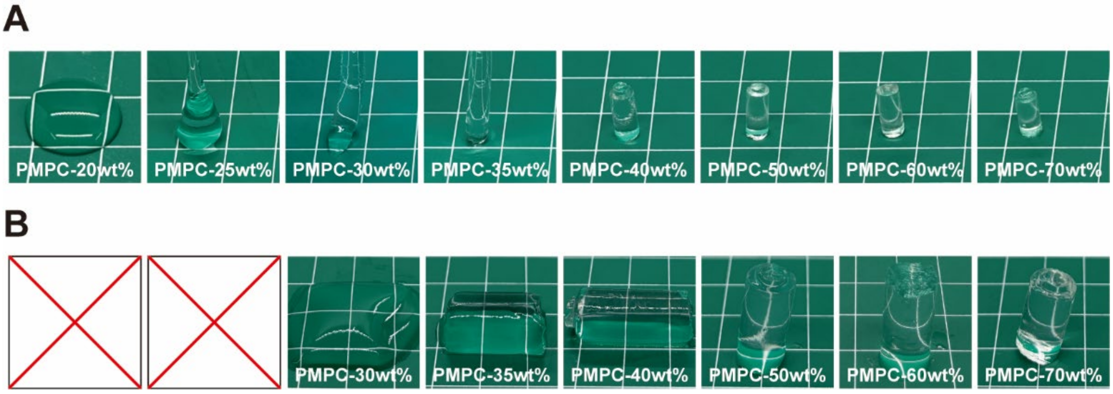

Figure S1. (A–B) Photographs of poly(2-methacryloyloxyethyl phosphorylcholine) PMPC samples immediately after photopolymerization at different MPC concentrations (A) and then after swelling in phosphate-buffered saline (PBS) for 7 days (B). No macroscopic gelation is observed at 20 wt%. The 25 wt% sample disintegrates during swelling.

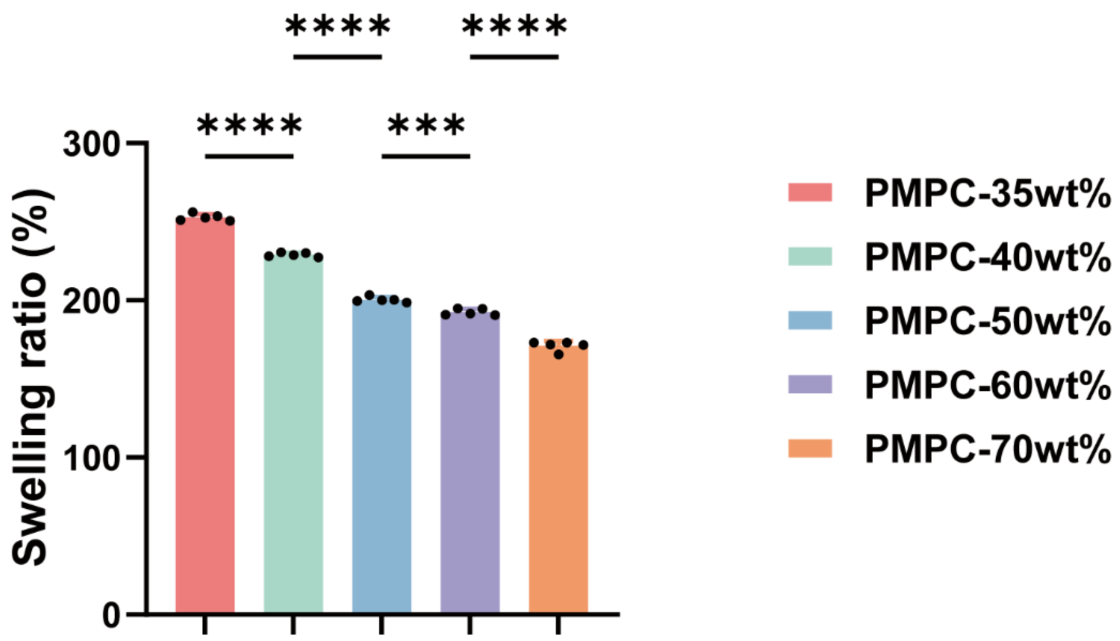

Figure S2. Photographs of PMPC samples immediately after photopolymerization at different MPC concentrations. Data are presented as mean ± SD (n = 5). *** p < 0.001, ****p < 0.0001; statistical analysis was performed using one-way analysis of variance (ANOVA) with Tukey's multiple-comparison test.

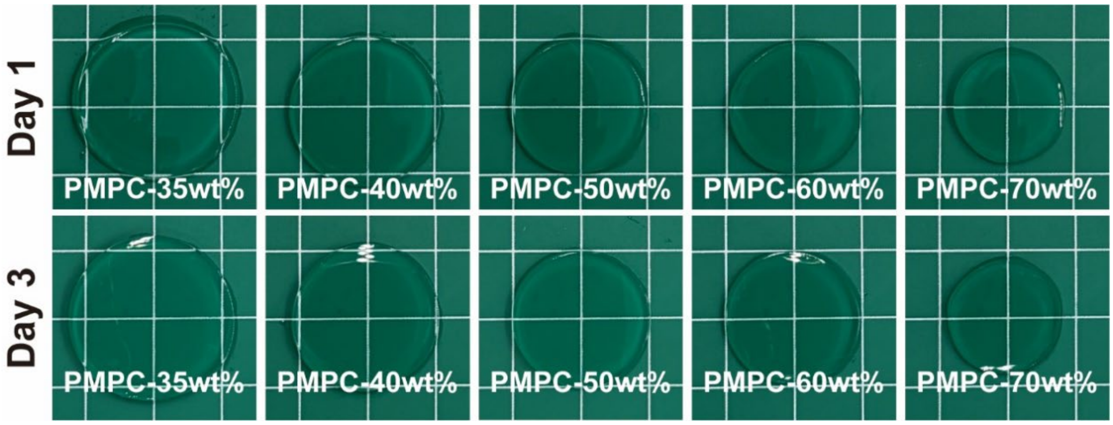

Figure S3. Photographs of PMPC hydrogel discs prepared at 35–70 wt% after swelling in PBS for 1 day and 3 days.

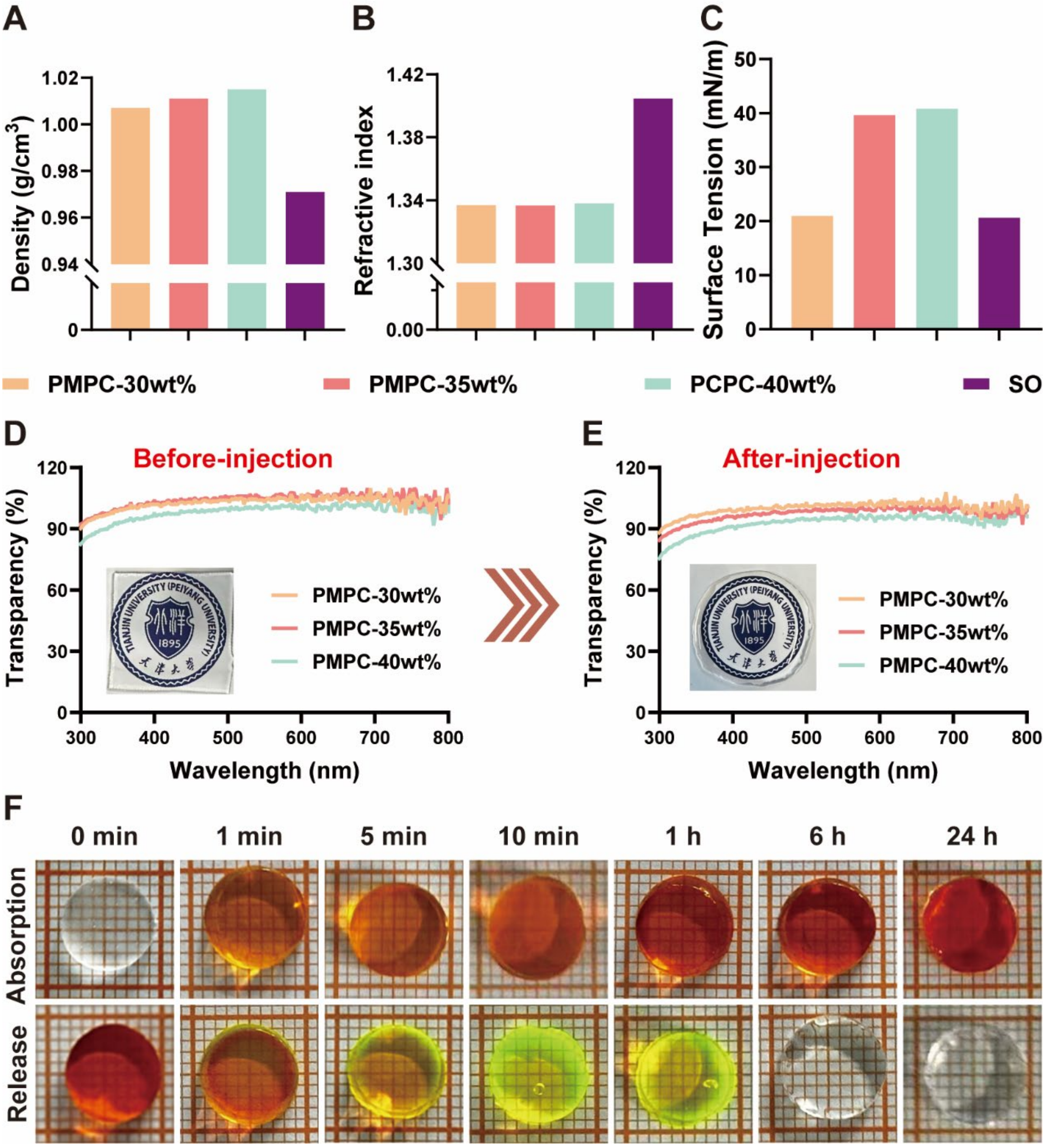

Figure S4. Physical characterization of PMPC hydrogels. (A–C) Density (A), refractive index (B), and surface tension (C) of PMPC hydrogels at 30–40 wt% were measured and compared with those of silicone oil (SO). (D and E) UV-vis transmittance spectra of PMPC hydrogels before injection (D) and after extrusion (E) through a 25-G needle. (F) Photographs showing sodium fluorescein permeation into the PMPC-35wt% hydrogel and subsequent release at different time points.

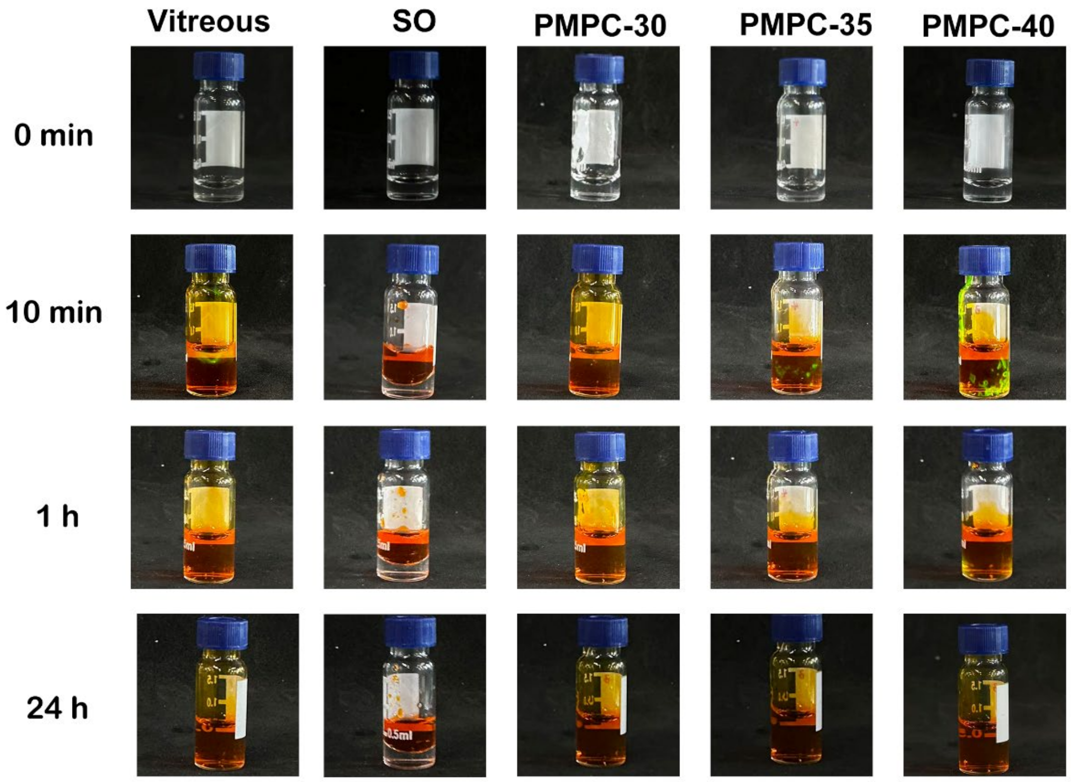

Figure S5. Permeability assessment of fluorescein sodium dye penetration of PMPC hydrogels, silicone oil (SO), and native vitreous at various time points.

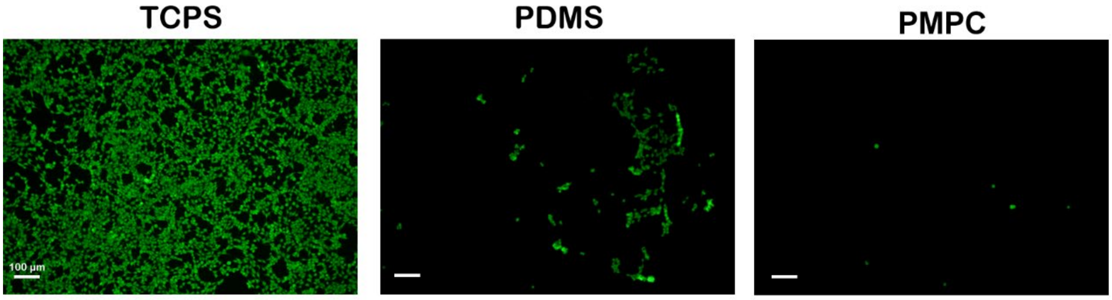

Figure S6. Representative images of R28 cells attached to tissue-culture polystyrene (TCPS), Polydimethylsiloxane (PDMS), and PMPC hydrogel surfaces (scale bar, 100 μm).

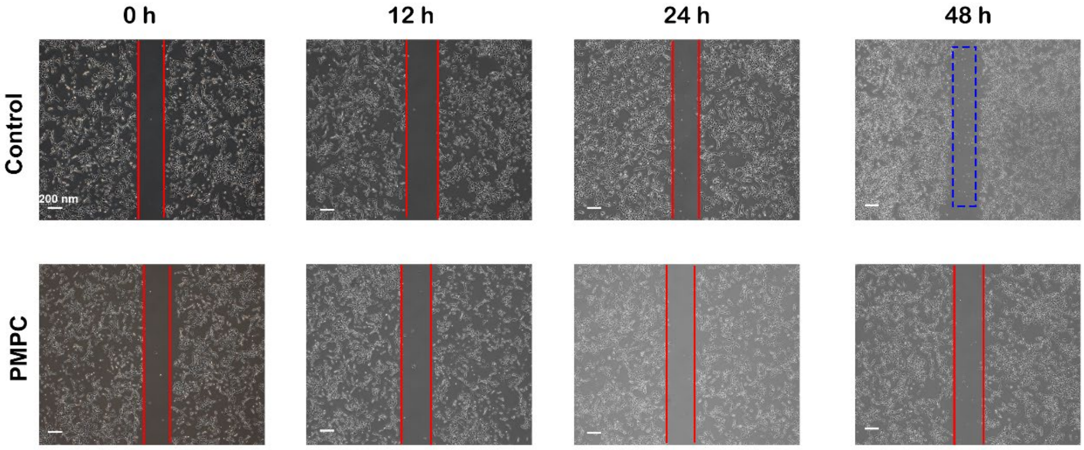

Figure S7. Representative migration images of R28 cells treated with PMPC hydrogel for 0, 12, 24, and 48 h (scale bar, 200 μm).

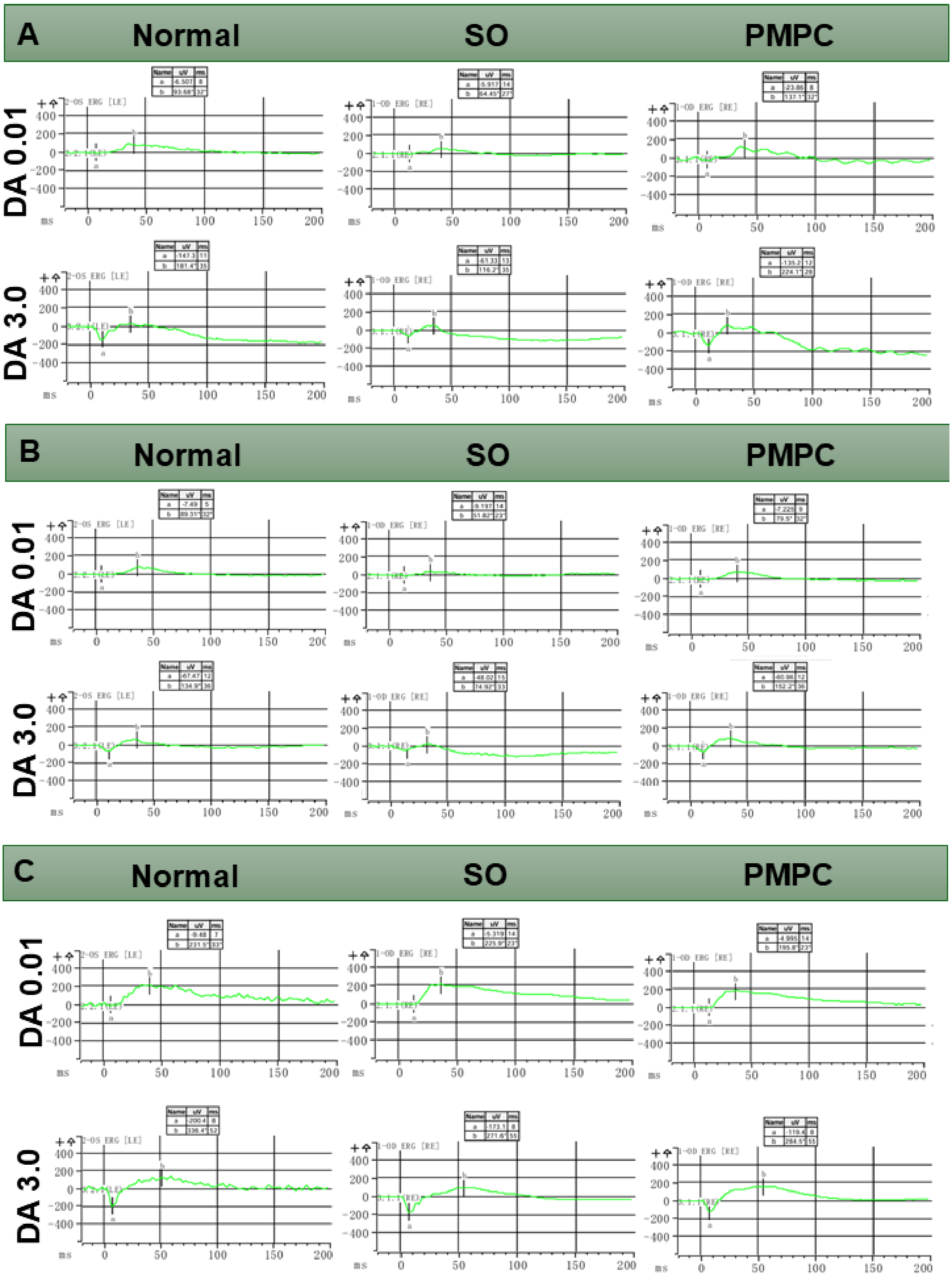

Figure S8. Electroretinogram (ERG) responses of Normal, SO, and PMPC groups at 1 month (A), 3 months (B), and 6 months (C) under different stimulation conditions: DA 0.01 and DA 3.0.

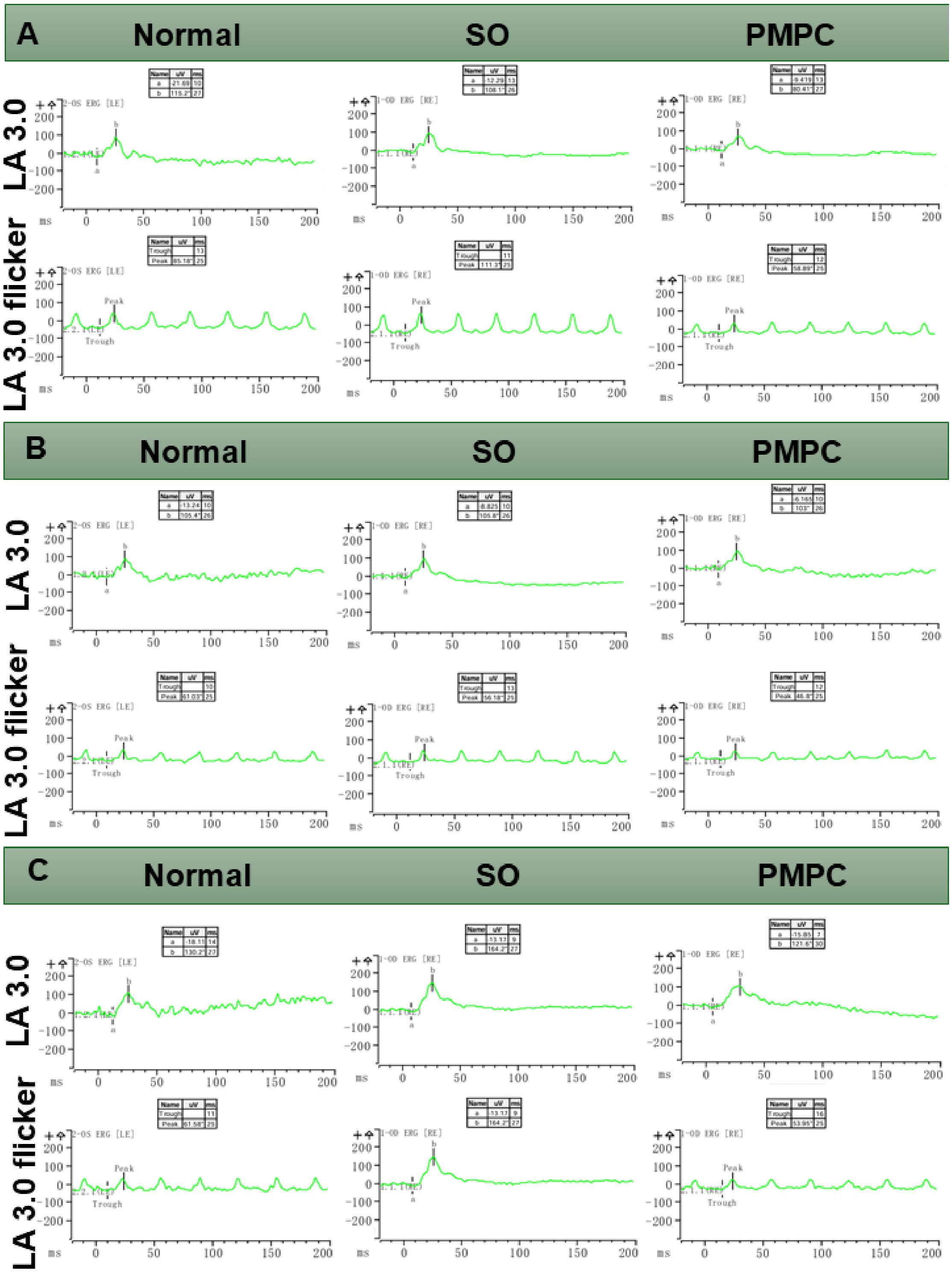

Figure S9. ERG responses of Normal, SO, and PMPC groups at 1 month (A), 3 months (B), and 6 months (C) under different stimulation conditions: LA 3.0 and LA 3.0 Flicker.

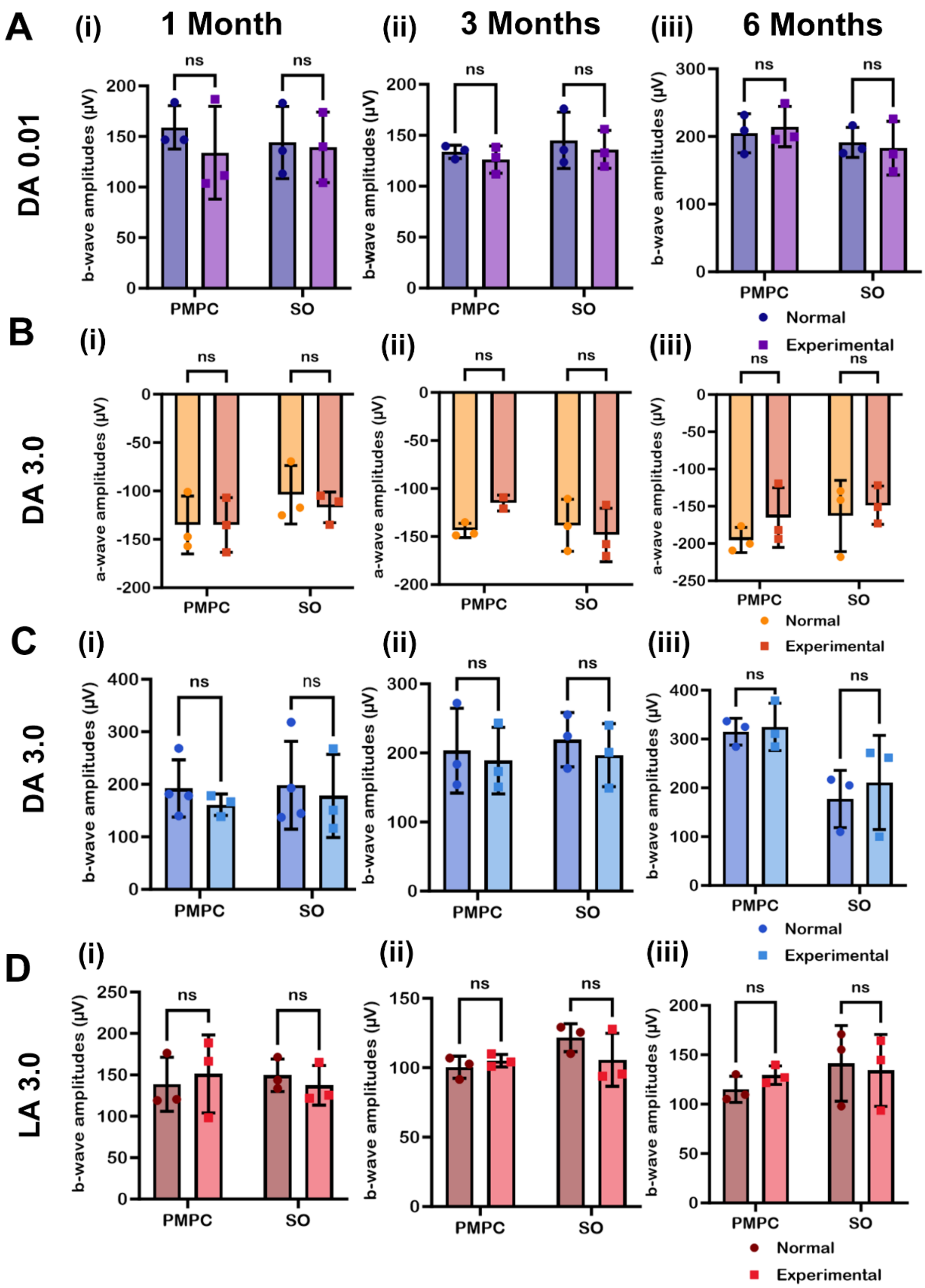

Figure S10. Functional assessment of rabbit retina by ERG. (A–D) ERG wave amplitudes for different groups and wave types at 1 month (i), 3 months (ii), 6 months (iii): DA 0.01 b-wave (A), DA 3.0 a-wave (B), DA 3.0 b-wave (C), and LA 3.0 b-wave (D). Data are presented as mean ± SD (n = 3). p values were calculated using an unpaired, two-tailed Student's t-test.

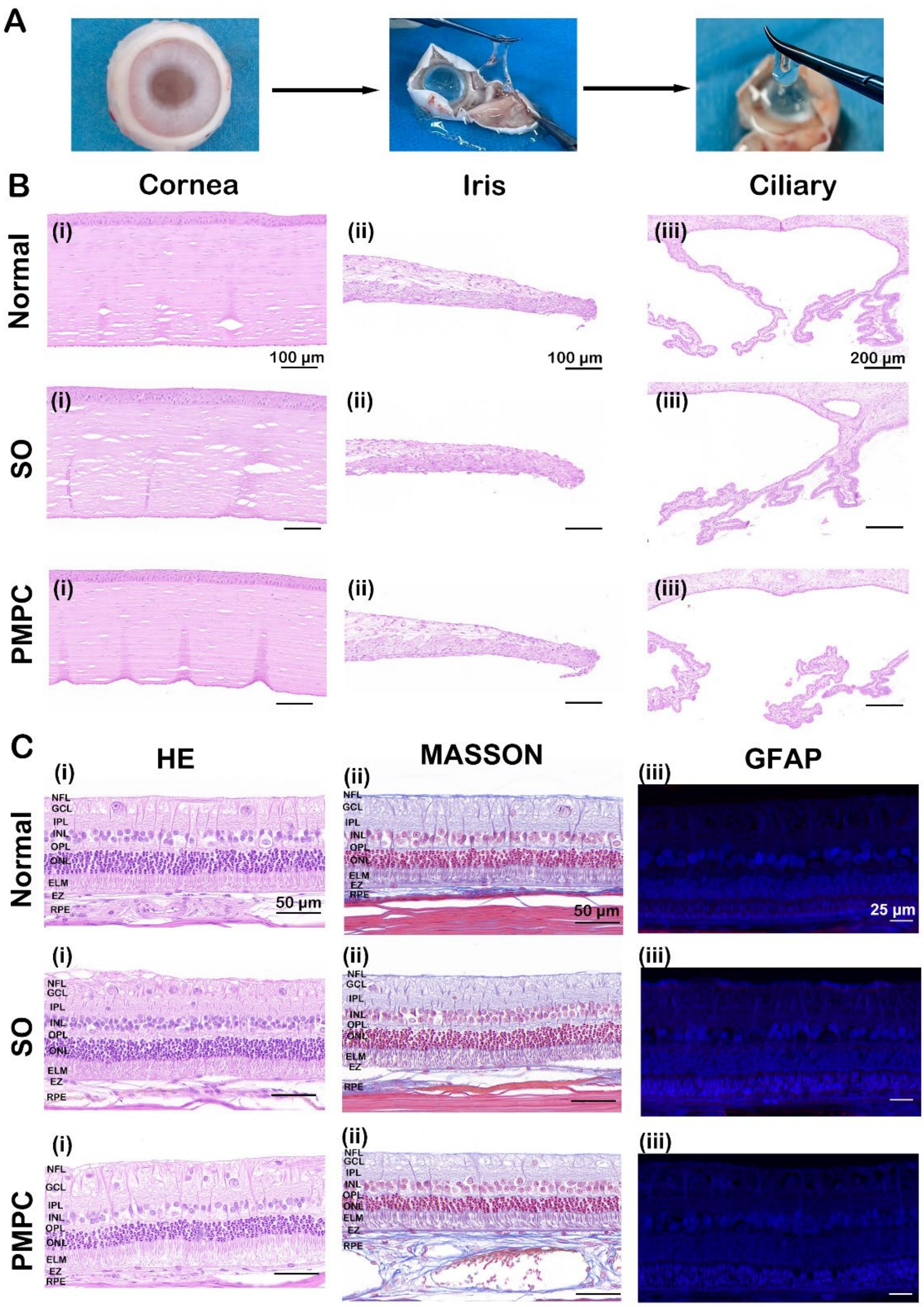

Figure S11. Histological evaluation of PMPC hydrogels as vitreous substitutes. (A) Gross photographs of the excised eye and the retrieved vitreous substitute after dissection, showing an intact gel phase that could be lifted with tweezers and retained its shape under its own weight. (B) Histological evaluation of rabbit eyes at 6 months post-implantation. (i–iii) H&E staining of cornea (i, scale bar, 100 µm), iris (ii, scale bar, 100 µm), and ciliary body (iii, scale bar, 200 µm). (C) Retinal evaluation at 6 months post-implantation. (i–iii) H&E staining (i, scale bar, 50 µm), Masson's trichrome staining (ii, scale bar, 50 µm), and GFAP immunostaining (iii, scale bar, 25 µm).

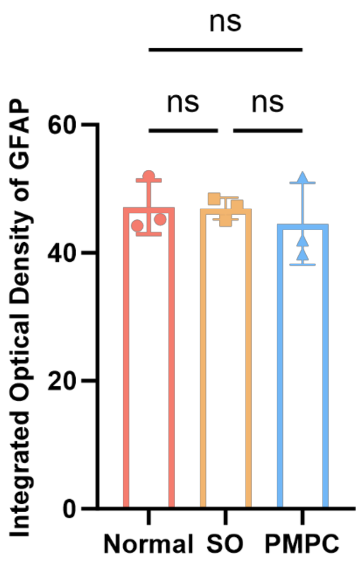

Figure S12. Quantitative analysis of GFAP immunostaining. Data are presented as mean ± SD (n = 3). p values were calculated using One-way ANOVA followed by Tukey's post hoc test. ns, not significant.

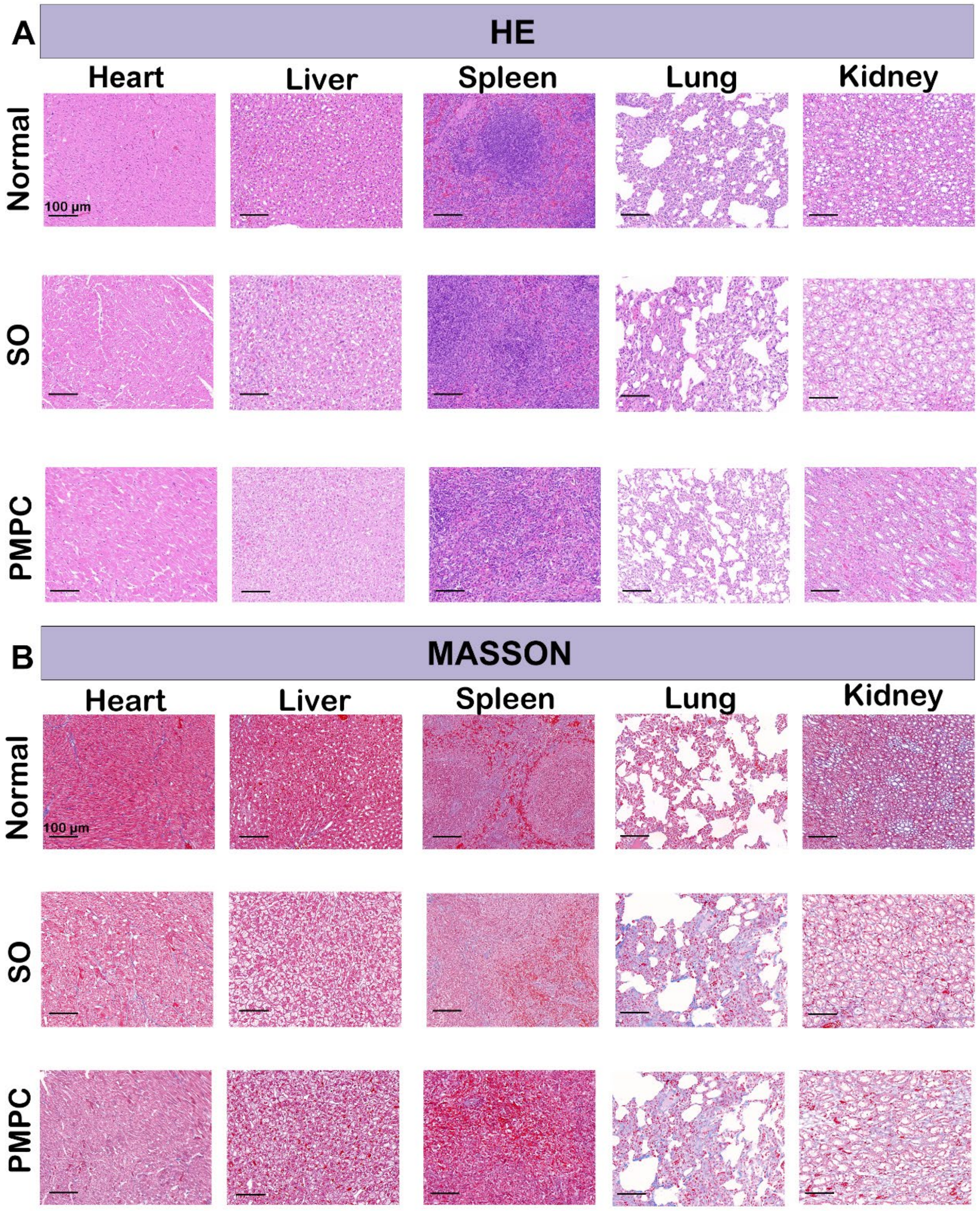

Figure S13. (A) Hematoxylin and Eosin (H&E) of rabbit tissues from heart, liver, spleen, lung, and kidney at 6 months. (B) Masson of rabbit tissues from the heart, liver, spleen, lung, and kidney at 6 months. The scale bars were 100 μm.

Supplementary Tables

Table S1. Equilibrium water content and density of PMPC hydrogels.

Sample	Density (g/cm^3)	Surface tension (mN/m)	Refractive index
Human vitreous	1.0053–1.0089[1]	47.8	1.3345–1.3348[2]
SO	0.96–0.98[3]	20.64	1.404[3]
PMPC-30wt%	1.007	20.99	1.3375
PMPC-35wt%	1.011	39.65	1.3372
PMPC-40wt%	1.015	40.81	1.3384

Supplementary Videos

Video S1. Vitrectomy procedure and PMPC hydrogel injection procedure.

Supplementary References

1. Confalonieri F, Josifovska N, Boix-Lemonche G et al. Vitreous substitutes from bench to the operating room in a translational approach: Review and future endeavors in vitreoretinal surgery. *Int J Mol Sci* 2023; **24**: 3342.
2. Gao Q-Y, Fu Y, Hui Y-N. Vitreous substitutes:Challenges and directions. *Int J of Ophthalmol* 2015; **8**, 437-440.
3. Giordano GG, Refojo MF. Silicone oils as vitreous substitutes. *Prog Polym Sci* 1998; **23**, 509-532.
